# Personalized cancer vaccine strategy elicits polyfunctional T cells and demonstrates clinical benefits in ovarian cancer

**DOI:** 10.1038/s41541-021-00297-5

**Published:** 2021-03-15

**Authors:** Janos L. Tanyi, Cheryl L.-L. Chiang, Johanna Chiffelle, Anne-Christine Thierry, Petra Baumgartener, Florian Huber, Christine Goepfert, David Tarussio, Stephanie Tissot, Drew A. Torigian, Harvey L. Nisenbaum, Brian J. Stevenson, Hajer Fritah Guiren, Ritaparna Ahmed, Anne-Laure Huguenin-Bergenat, Emese Zsiros, Michal Bassani-Sternberg, Rosemarie Mick, Daniel J. Powell, George Coukos, Alexandre Harari, Lana E. Kandalaft

**Affiliations:** 1 Ovarian Cancer Research Center, Abramson Cancer Center, Perelman School of Medicine, University of Pennsylvania, Philadelphia, PA USA; 2 Department of Oncology, Lausanne University Hospital (CHUV), Ludwig Institute for Cancer Research, University of Lausanne, Lausanne, Switzerland; 3 Center of Experimental Therapeutics, Department of Oncology, Lausanne University Hospital (CHUV), Lausanne, Switzerland; 4 Institute of Animal Pathology, COMPATH, Vetsuisse Faculty, University of Bern, Bern, Switzerland; 5 School of Life Sciences, Ecole Polytechnique Fédérale de Lausanne, Lausanne, Switzerland; 6 Department of Radiology, Hospital of the University of Pennsylvania, Philadelphia, PA USA; 7 Department of Biostatistics and Epidemiology, Perelman School of Medicine, University of Pennsylvania, Philadelphia, PA USA

**Keywords:** Cancer, Immunology, Cancer immunotherapy, Cancer therapy, Tumour immunology

## Abstract

T cells are important for controlling ovarian cancer (OC). We previously demonstrated that combinatorial use of a personalized whole-tumor lysate-pulsed dendritic cell vaccine (OCDC), bevacizumab (Bev), and cyclophosphamide (Cy) elicited neoantigen-specific T cells and prolonged OC survival. Here, we hypothesize that adding acetylsalicylic acid (ASA) and low-dose interleukin (IL)-2 would increase the vaccine efficacy in a recurrent advanced OC phase I trial (NCT01132014). By adding ASA and low-dose IL-2 to the OCDC-Bev-Cy combinatorial regimen, we elicited vaccine-specific T-cell responses that positively correlated with patients’ prolonged time-to-progression and overall survival. In the ID8 ovarian model, animals receiving the same regimen showed prolonged survival, increased tumor-infiltrating perforin-producing T cells, increased neoantigen-specific CD8^+^ T cells, and reduced endothelial Fas ligand expression and tumor-infiltrating T-regulatory cells. This combinatorial strategy was efficacious and also highlighted the predictive value of the ID8 model for future ovarian trial development.

## Introduction

T cells are essential for controlling cancer progression. Prior studies in ovarian cancer (OC) patients consistently reported a correlation between CD3^+^ tumor-infiltrating lymphocytes (TILs) and overall survival (OS)^1–4^. This observation provides a rationale for using immunotherapies to mobilize endogenous antitumor T cells. We previously demonstrated that vaccinating OC patients with autologous dendritic cells (DCs) pulsed with oxidized autologous whole-tumor lysate (termed OCDC) elicited T cells against shared tumor-associated antigens, including HER-2/neu, MUC1, and WT1 and private neoantigens^5,6^.

OC harbors immunosuppressive mechanisms that hinder antitumor immunity and combinatorial therapeutic strategies are needed to overcome them. In the OC tumor microenvironment (TME), vascular endothelial growth factor (VEGF) and T-regulatory (Treg) cells are important immunosuppressive barriers that could drive tumor angiogenesis, as well as prevent effective homing, expansion, and function of tumor-specific T cells^7^. Most OCs express VEGF and increased sera VEFG levels have been correlated with poor survivals^8–10^. Treg cells suppress tumor-specific T cells and contribute to tumor progression^11,12^. They also accumulated in advanced OCs^12^. By targeting VEGF and Treg cells with bevacizumab (Bev) and cyclophosphamide (Cy), respectively, we previously demonstrated that OCDC used in combination with these agents led to enhanced OC survivals (NCT01132014; www.clinicaltrials.gov)^6^. Inhibiting VEGF could also lead to “normalization” of tumor vasculature, improving oxygen, nutrient, and chemotherapy delivery to increase tumor toxicity and reduce ascites fluid formation^13,14^.

Here, we sought to enhance the antitumor T-cell responses of OCDC by incorporating acetylsalicylic acid (ASA) and low-dose interleukin (IL)-2 in the combinatorial strategy. ASA, a classical nonsteroidal anti-inflammatory drug (NSAID), is widely used for treating fever, pain, and inflammatory diseases^15^. It irreversibly inhibits cyclooxygenase (COX)1 and COX2 enzymes and thereby inhibits prostaglandin E2 (PGE2) synthesis. Long-term ASA use has been associated with reduced cancer incidences and mortalities, including in ovarian, colorectal, breast, and esophageal cancers^16–18^. We previously demonstrated that the OC tumor endothelium acted as a potent immune barrier by expressing the death mediator Fas ligand (FasL/CD95L) that preferentially killed CD8^+^ T cells, and such FasL expression was cooperatively induced by tumor-derived VEGF, IL-10, and PGE2^19^. By inhibiting VEGF and PGE2 with ASA and an anti-VEGF blocking antibody, respectively, the tumor endothelial FasL expression was attenuated and that facilitated a substantial infiltration of tumor-suppressing CD8^+^ TILs in the ID8 OC tumor model^19^. In human OC, COX1 and COX2 overexpression was significantly associated with lower numbers of CD8^+^ TILs and worse prognosis^20,21^. Hence, ASA and anti-VEGF-blocking antibodies are useful in modulating OC tumor endothelial FasL expression. IL-2 is a major T-cell growth factor that can assist in the in vivo expansion of antigen-specific T cells and lymphokine-activated killer (LAK) cells^22–24^. As OC patients exhibit spontaneous antitumor immune responses, IL-2 therapy could help to activate preexisting immunity or enhance immunomodulatory therapy. In OC, low-dose subcutaneous IL-2 in combination with 13-cis-retinoic acid could improve progression-free survival (PFS) and OS^25^. VEGF was also decreased, and the CD4^+^/CD8^+^ ratio improved in patients^25^. IL-2 could also increase adoptive T-cell therapies and DC vaccine effectiveness^26–29^. In phase II metastatic melanoma study, combinatorial treatment with DC vaccine (peptide-pulsed or whole-tumor lysate), IL-2 and metronomic Cy and Celecoxib (a COX2 inhibitor) was safe, and 16 out of 28 (57%) patients experienced stable diseases (NCT00197912; www.clinicaltrials.gov)^30^. Thus, IL-2 could potentially benefit the proliferation and function of effector T cells elicited by OCDC in this study.

We report here the promising results of a combinatorial strategy that comprised OCDC, Bev, ASA, Cy, and low-dose IL-2 in a phase I trial with recurrent advanced OC patients (NCT01132014; www.clinicaltrials.gov), and in the syngeneic mouse ID8 OC model. We demonstrated that this combinatorial strategy was effective and elicited polyfunctional (IFN-γ, TNF-α, granzyme, and perforin) vaccine-specific T-cell responses in OC patients that correlated with prolonged time-to-progression and OS. In the ID8 model, animals receiving the same regimen showed prolonged survival, increased CD3^+^ and CD8^+^ TILs, increased neoantigen-specific CD8^+^ T-cell responses that were correlated with reduced tumor burden, and a systemic effect with elevated T helper (Th)1-polarizing plasma chemokines. Particularly, elevated sera CXCL9 was positively correlated with increased CD3^+^ and CD8^+^ TILs. The similar trend observed in the phase I OC trial and in the ID8 model highlighted the relevance of using the ID8 model for OC clinical translation.

## Results

### The addition of ASA and low-dose IL-2 to an OCDC-Bev-Cy combinatorial regimen induced an immune response and prolonged time-to-progression and OS in recurrent OC patients

**Fig. 1:**
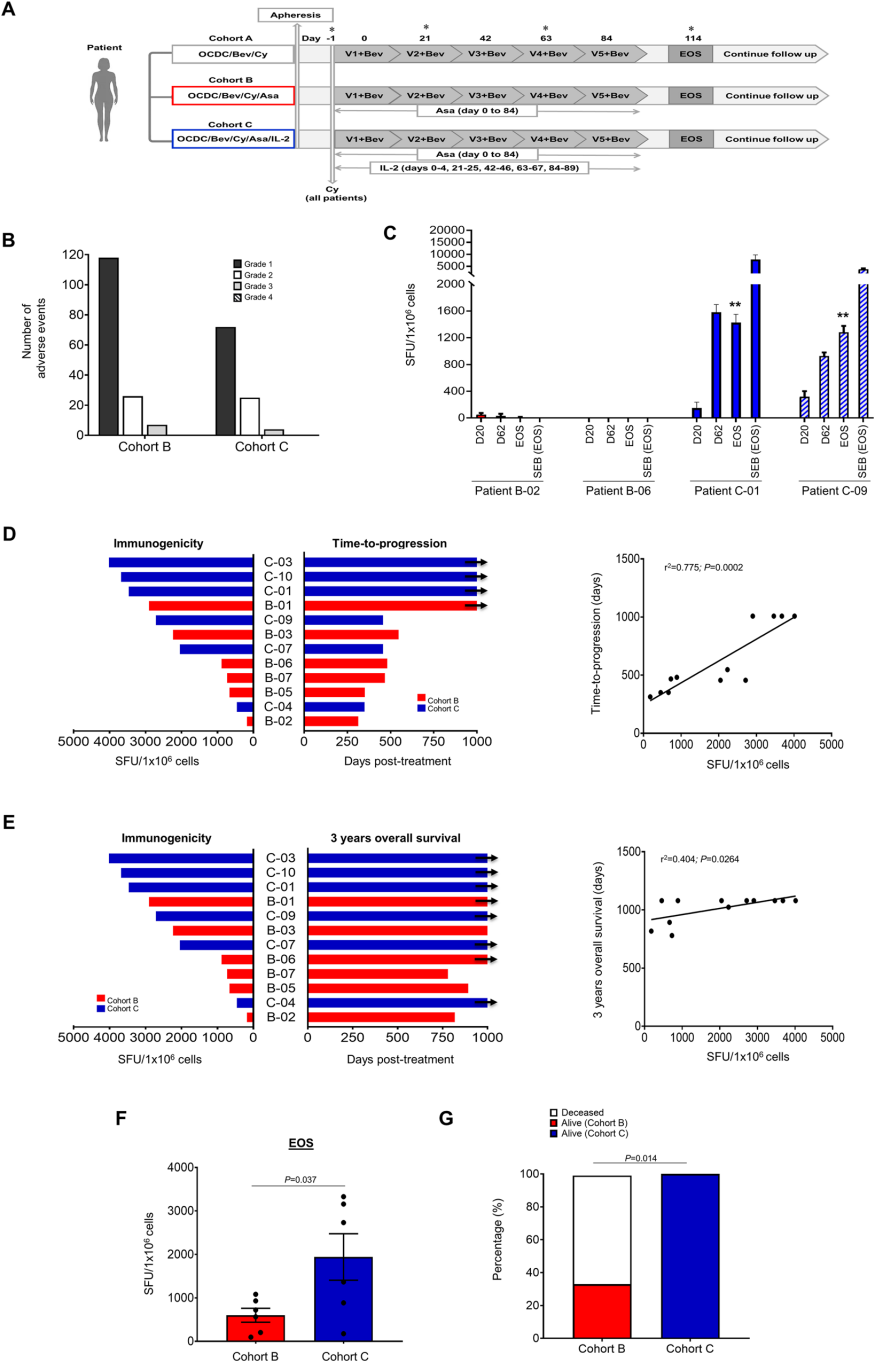
Adding ASA and low-dose IL-2 to OCDC-Bev-Cy combinatorial regimen prolonged OC survival. **A** OC patients were enrolled in phase I, a single-center study (ten patients/cohort). Cohort A received OCDC, Bev, and Cy. Cohort B received additional daily oral ASA (325 mg of enteric-coated aspirin) from day 0 to 84, while Cohort C received oral ASA and subcutaneous low-dose IL-2 (2MIU/dose given for 5 consecutive days following each OCDC). Patients were followed up for 3 years. Asterisks indicated time points that the patient’s PBMCs were obtained (day −1 (pre-vaccine), 20, 62, and 114) for immune analysis. **B** Number of adverse events (AEs) in patients during treatment. **C** OCDC vaccine-specific responses in Cohort B and C patients. PBMCs were stimulated ex vivo with OCDC or media as background control. The results were subtracted from pre-vaccine readout and reported as a spot-forming unit (SFU)/1 × 10^6^ cells±standard error of the mean (SEM). **D** Number of OCDC vaccine-specific T cells detected by IFN-γ ELISpot and time-to-progression in Cohort B and C patients. Left panel, patients were ranked based on the magnitude of OCDC vaccine-specific T-cell responses detected during treatment (background subtracted) (*n* = 6 patients per cohort]. Right panel, linear regression analysis indicated a positive correlation between increasing vaccine immunogenicity and increasing time-to-progression (*r*^2^ = 0.775*; P* = 0.0002). **E** Number of OCDC vaccine-specific T cells detected by IFN-γ ELISpot and 3-years overall survival (OS) in Cohort B and C patients. Left panel, patients were ranked as in (**D**). Right panel, linear regression analysis indicated a positive correlation between increasing vaccine immunogenicity and increasing 3-years OS (*r*^2^ = 0.404*; P* = 0.0264). **F** Number of OCDC vaccine-specific T cells detected in Cohort B and C patients at EOS (Student’s *t* test; *P* = 0.0264). **G** Stacked bars showing % of Cohort B and C patients alive (solid red and blue, respectively) or deceased (white) at 3-years OS. *P* value determined with Fisher’s exact test.

We previously demonstrated that the addition of Cy to OCDC + Bev significantly increased antigen-specific T-cell responses and OS rate at 2 years^6^. In this study, we investigated if adding ASA and low-dose IL-2 could further enhance antitumor T-cell responses in a phase I trial of 30 platinum-pretreated, immunotherapy-naive recurrent advanced OC patients (NCT01132014) (ten patients per cohort). Treatment combinations and schedules are shown in Fig. 1A. Patients were followed up to 3 years and evaluated for time-to-progression and 3-years OS from the initiation of OCDC-Bev-Cy combinatorial regimens (i.e., day 0; Fig. 1A). Most patients had received several prior lines of chemotherapy, and their clinicopathologic characteristics, treatment details, and 3-year OS outcome were described in Table 1. All patients received intravenous Cy (200 mg/m^2^) followed by OCDC (5–10 × 10^6^ DCs/dose given intranodally every 3 weeks, five doses in total) plus intravenous Bev (anti-VEGF blocking antibody, 15 mg/kg, given on the same day as OCDC) on the next day every 3 weeks. Cohort B and C received additional oral ASA (325 mg of enteric-coated aspirin from the day of first OCDC to day 84 or as tolerated). Cohort C also received subcutaneous low-dose IL-2 (2MIU/dose given for 5 consecutive days following each OCDC). As Cohort A was previously described^6^, this study reported findings in Cohorts B and C patients.

**Table 1:** Ovarian cancer (OC) patients’ characteristics and treatment details.

Patient ID	Age	Median age	Cancer type	Stage	Number of prior chemotherapies	Number of prior recurrences	Secondary debulking outcome*	Residual disease	Number of OCDC vaccines received	Outcome at 3-year OS
B-01	53	59 ± 9	Ovarian	IIIC	2	1	Optimal	No	20	Alive
B-02	54		Ovarian	IV	5	2	Sub	Yes	8	Deceased
B-03	65		Ovarian	IV	3	1	Optimal	No	11	Deceased
B-04	49		Ovarian	IIIC	3	1	Sub	Yes	5	Deceased
B-05	44		Ovarian	IIIC	2	2	Optimal	No	11	Deceased
B-06	61		Fallopian	IIC	2	1	Optimal	Yes	11	Alive
B-07	74		Ovarian	IIIC	4	2	Optimal	No	17	Deceased
B-08	57		Ovarian	IIIC	3	1	Sub	Yes	5	Alive
B-09	69		Ovarian	IIIC	4	1	Optimal	No	5	Deceased
B-10	67		Ovarian	IIIC	3	1	Optimal	Yes	5	Alive
C-01	60	56 ± 11	Ovarian	IIIC	2	1	Optimal	Yes	30	Alive
C-02	57		Ovarian	IV	3	1	Optimal	Yes	5	Deceased
C-03	54		Ovarian	IIIC	2	1	Sub	Yes	28	Alive
C-04	72		Ovarian	IIIC	3	1	Sub	Yes	11	Alive
C-05	73		Peritoneal	IIIC	2	1	Optimal	No	2	Alive
C-06	37		Ovarian	IIIC	5	1	Sub	Yes	3	Alive
C-07	46		Ovarian	III	2	1	Optimal	No	8	Alive
C-08	50		Ovarian	III	6	2	Sub	Yes	3	Deceased
C-09	52		Ovarian	IIIC	5	1	Optimal	Yes	8	Alive
C-10	65		Peritoneal	IIIC	6	1	Optimal	Yes	9	Alive

*Sub indicated a suboptimal secondary debulking.

Cohort B and C treatment regimens were safe and well-tolerated. Few grade 3 adverse events (AEs) such as hypotension, proteinuria, vomiting, decreased absolute neutrophil count, thrombocytosis, and skin infection were observed and were effectively managed. We did not observe any grade 4 AEs (Fig. 1B). The commonest grade 3 AE in both cohorts was hypotension (5 occurrences). First, we investigated the kinetics of OCDC vaccine-elicited T cells at day 20-, day 62-, and end-of-study (EOS). We observed that Cohort C patients C-01 and C-09, who showed prolonged time-to-progression and/or 3-years OS, demonstrated a persistent increase in the number of IFN-γ-secreting vaccine-specific T cells at EOS that was significantly higher than at pre-vaccine (day −1) (Fig. 1C; ** indicated that *P* < 0.0005 comparing pre-vaccine and EOS T cells in the same patient). Less potent vaccine immunogenicity was observed in Cohort B patients B-02 and B-06 who showed shorter time-to-progression and 3-years OS (Fig. 1C). Next, we sought to determine if there was a positive correlation between the increasing number of IFN-γ-secreting vaccine-specific T cells elicited during treatment and a prolonged time-to-progression in Cohorts B and C patients. Of the 12 patients (6 from each cohort) whom we had available PBMCs for evaluation, we compared the highest number of vaccine-specific T cells elicited (i.e., immunogenicity) during treatment in each patient and the patient’s corresponding time-to-progression. We demonstrated that patients who displayed a higher number of IFN-γ-secreting vaccine-specific T cells also experienced a longer time-to-progression (*r*^2^ = 0.775; *P* = 0.0002) (Fig. 1D, right panel). Three out of six Cohort C patients (Fig. 1D, left panel, blue bars) as compared to one out of six Cohort B patients (Fig. 1D, left panel, red bars) experienced a prolonged time-to-progression of ≥1000 days (indicated with black arrows). We also demonstrated a significant positive correlation between OCDC immunogenicity and a prolonged 3-years OS in these patients (*r*^2^ = 0.404; *P* = 0.0264) (Fig. 1E, right panel). Of the 12 patients analyzed, all 6 Cohort C patients remained alive at 3-years OS when compared to 2 (Patients B-01 and B-06) out of the 6 Cohort B patients (Fig. 1E right panel; black arrows indicated that patients were alive at 3-years, and Fig. 1G (*P* = 0.014)). Overall, these Cohort C patients showed a significantly higher number of vaccine-specific T cells at EOS than Cohort B patients (*P* = 0.037) (Fig. 1F). In summary, 80% (eight of ten) of Cohort C patients remained alive at 3-years OS when compared to Cohort A (40%; four of ten) and B (40%; four of ten) patients (Table 1; Cohort A data not shown). Hence, the results demonstrated that the addition of both ASA and low-dose IL-2 to an OCDC-Bev-Cy combinatorial regimen increased vaccine immunogenicity and clinical benefit in OC patients.

### The addition of ASA and low-dose IL-2 to an OCDC-Bev-Cy combinatorial regimen elicits polyfunctional T-cell responses in OC patients

**Fig. 2:**
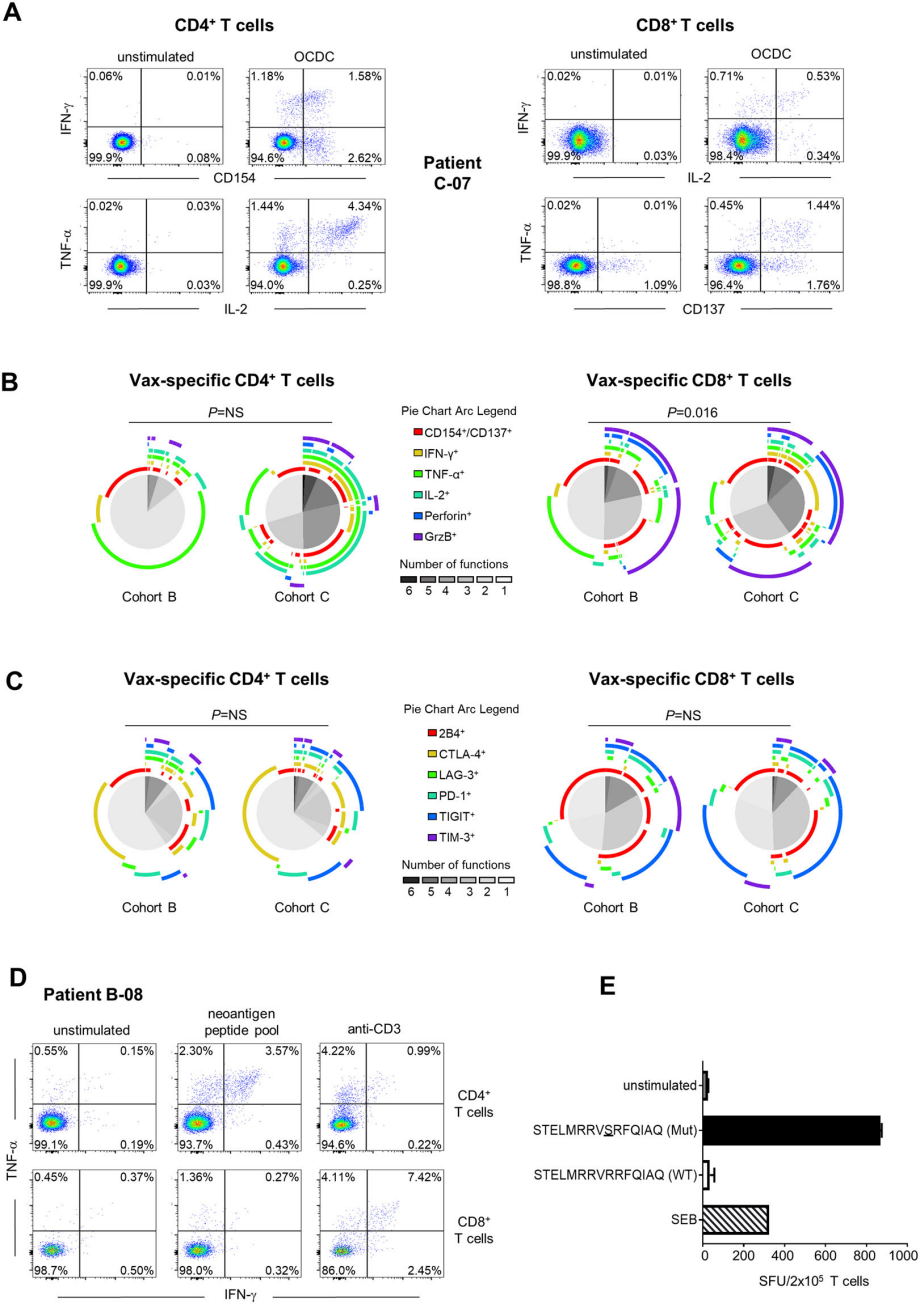
The addition of ASA and low-dose IL-2 to an OCDC-Bev-Cy combinatorial regimen elicits polyfunctional T-cell responses in OC patients. **A** Examples in patient C-07 of polyfunctional OCDC vaccine-specific CD4^+^ and CD8^+^ T-cell responses elicited. **B** Cumulative functional profiling of OCDC vaccine-specific CD4^+^ and CD8^+^ T-cell responses where cytokine-producing T cells (as shown in (**A**)) in response to ex vivo OCDC stimulation were evaluated for (co)expressions of different functional markers as illustrated by the colored arcs. Pie charts represented in shades of gray depicting the number of markers coexpressed by OCDC vaccine-specific T cells in Cohort B and C patients (*n* = 6 patients per cohort). **C** Cytokine-producing CD4^+^ and CD8^+^ T cells following ex vivo OCDC stimulation were evaluated for (co)expression of the different activation/exhaustion markers as illustrated by the colored arcs. Pie charts in shades of gray depicted the number of markers coexpressed on the OCDC vaccine-specific T cells in patients. *N* = 6 patients. **D** Identification of neoepitope-specific CD4^+^ and CD8^+^ T-cell responses in Cohort B-08 patient. Neoepitopes from the peptide pool were described in Supplementary Table S1. **E** Bar charts showed the validation of the IFN-γ ELISpot T-cell response against the (STELMRRVSRFQIAQ [Mut]) neoepitope as well as the lack of response against the wild-type (STELMRRVRRFQIAQ [WT]) cognate peptide. *P* values for SPICE pies were determined with Wilcoxon rank-sum test. *P* < 0.05 was considered significant, while *P* > 0.05 was not significant (NS).

Having observed that Cohort C regimen induced a stronger OCDC vaccine-specific T-cell response than Cohort B regimen, we sought to determine if the responding CD4^+^ and CD8^+^ T cells were qualitatively different between these two cohorts at EOS. Showing Cohort C-07 patient as an example response (Fig. 2A), we demonstrated that CD4^+^ and CD8^+^ T cells elicited in both cohorts were polyfunctional, i.e., simultaneously displayed a high number of functions including expression of IFN-γ, IL-2, TNF-α, CD137, and cytolytic molecules perforin and granzyme B (Fig. 2B). Particularly, vaccine-specific CD8^+^ T cells elicited by Cohort C regimen were significantly more polyfunctional than those elicited by Cohort B regimen (*P* = 0.016) (Fig. 2B, right panel and Supplementary Fig. 1A showing gating strategy). We further demonstrated that significant polyfunctional vaccine-specific CD4^+^ and CD8^+^ T-cell populations were elicited in Cohort C patients and not in Cohort B patients; these T cells predominantly expressed a combination of perforin and granzyme B, IFN-γ, and TNF-α (Supplementary Fig. 1B, C).

Next, we evaluated the expression of activation/exhaustion markers including 2B4 (CD244), CTLA4, LAG3, PD1, TIGIT, and TIM3 on vaccine-specific CD4^+^ and CD8^+^ T cells between Cohorts B and C, and did not observe any significant differences (Fig. 2C and Supplementary Fig. 2A showing gating strategy). We also found no significant differences in the CD4^+^CD25^+^FOXP3^+^ Treg cell populations and their expression markers in the two cohorts (Supplementary Fig. 2B showing gating strategy and Supplementary Fig. S2C, D). We did observe trends toward a higher proportion of IL-17^+^CD4^+^ T cells, as well as a higher frequency of Ki67-expressing CD8^+^ T cells elicited by OCDC vaccine in Cohort C (Supplementary Fig. 2E, F). Finally, we sought to identify immune responses against tumor neoepitopes in an OC patient (B-08) whom we had available tumor and PBMC materials. To this end, HLA-restricted neoepitopes were predicted as previously described^6,31^ (Supplementary Table 1). We detected both CD4^+^ and CD8^+^ T-cell responses against pools of private neoepitopes (Fig. 2D). We further validated a positive CD4^+^ T-cell response to a putative predicted HLA Class II-restricted neoepitope combined with the lack of reactivity against the wild-type peptide sequence (Fig. 2E). This observation indicated that both HLA class I and II-restricted neoepitopes were elicited by OCDC vaccination. We demonstrated that adding both ASA and low-dose IL-2 to an OCDC-Bev-Cy combinatorial regimen increased the proportion of polyfunctional vaccine-specific CD8^+^ T cells in OC patients.

### The addition of ASA and low-dose IL-2 to an OCDC-Bev-Cy combinatorial regimen prolongs survival and enhanced tumor infiltration of cytolytic CD3^+^ and CD8^+^ T cells in an OC mouse model

**Fig. 3:**
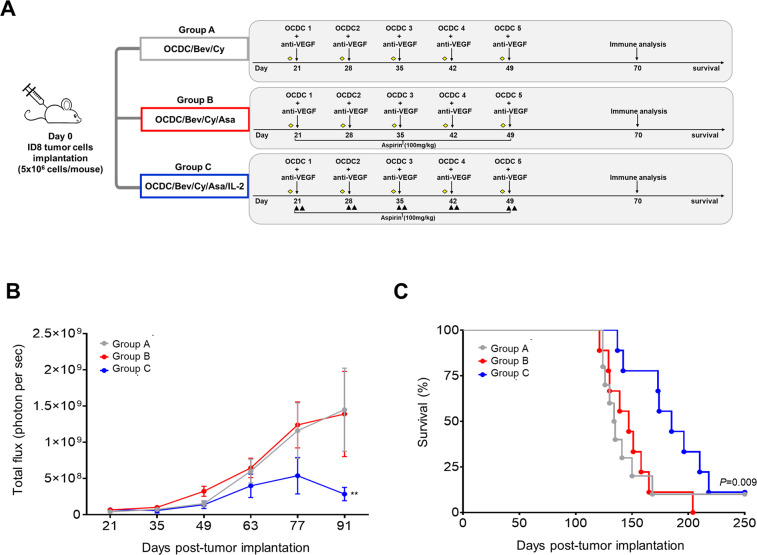
The addition of ASA and low-dose IL-2 to an OCDC-Bev-Cy combinatorial regimen prolongs survival and reduces tumor burden in the ID8 ovarian tumor model. **A** Syngeneic 8–10 weeks old female C57BL/6 mice were implanted i.p. with ID8 tumor cells (5 × 10^6^ cells/animal) and randomized into Group A, B, or C. All animals received Cy (40 mg/kg, indicated as diamonds, given i.p.) before each OCDC, and anti-VEGF blocking antibody (2 mg/kg, clone B20-4.1.1, mouse IgG2a, given intraperitoneally [i.p.]) together with each OCDC. Group B and C received ASA via drinking water (100 mg/kg). Group C also received recombinant mouse low-dose IL-2 (15 µg/kg, given i.p.) on 2 consecutive days following OCDC vaccination. Five weekly OCDCs were given intradermally in the groin region (1 × 10^6^ DCs/50 µl sterile PBS/animal). **B** Quantification of tumor burden with bioluminescence imaging. Results were reported as total flux (photons per second). The tumor burden of Group C was significantly lower when compared to Group A (*P* = 0.008) and B (*P* = 0.004). One-way ANOVA and Tukey’s multiple comparisons test for significance were performed. **C** Comparison of the survival curves of animals receiving different regimens (*P* = 0.009; Log-rank Mantel–Cox test). Group C showed the longest median OS of 185 days. In all the analyses, *P* < 0.05 was considered significant. The data were representative of three separate experiments. Total *n* = 9 animals per group.

We used the syngeneic ID8 OC model to investigate if observations from our phase I OC patients through the addition of ASA and low-dose IL-2 to OCDC, Bev, and Cy (hereafter referred to as “baseline treatment”^6^) could be replicated in mouse avatars. Tumor-bearing animals were randomized into three treatment groups closely mimicking the regimens proposed in the phase I OC clinical trial (Fig. 3A). All animals received the baseline treatment. Group C animals receiving additional ASA and low-dose IL-2 demonstrated a significantly lower tumor burden that decreased over time compared to Groups A (*P* = 0.008) and B (*P* = 0.004) (Fig. 3B). Group C animals also demonstrated the longest median survival (185 days, *P* = 0.009) (Fig. 3C, blue line)), while Group B animals which received ASA without low-dose IL-2 showed no significant improvement in survival over Group A (median survival of 147 days vs. 134.5 days) (Fig. 3C, gray and red lines). Moreover, Group C animals displayed a significantly prolonged OS, whereas animals treated with monotherapies exhibited poor OS that was similar to the PBS-treated control group (Supplementary Fig. 3). Similar to OC patients, the addition of both ASA and low-dose IL-2 significantly increased the overall efficacy of the baseline treatment in the treated animals.

**Fig. 4:**
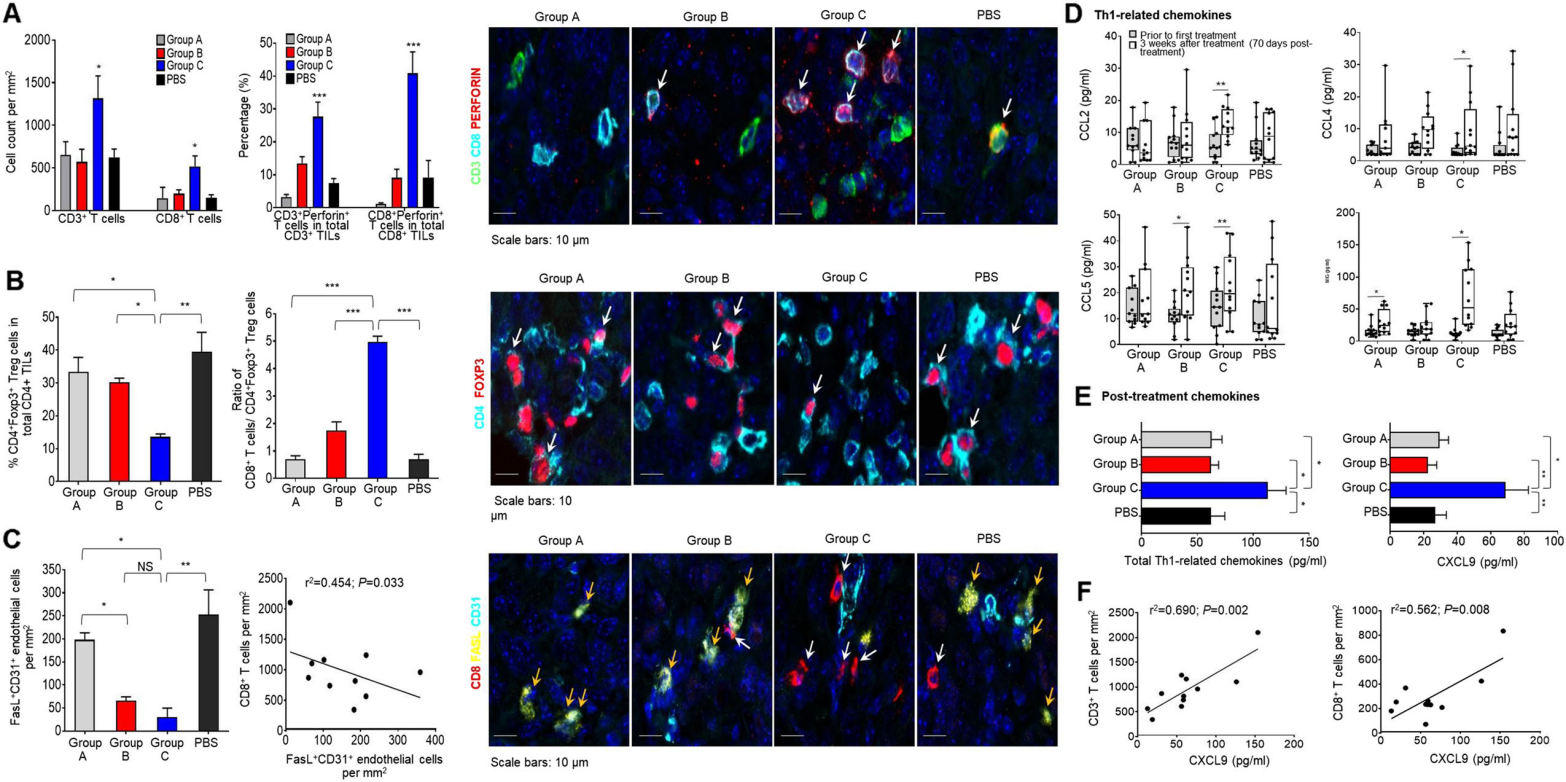
Adding ASA and low-dose IL-2 to OCDC-Bev-Cy combinatorial regimen enhances CD3^+^ and CD8^+^ TILs in the ID8 OC model. Eight-to-ten-week-old female C57BL/6 mice (5 × 10^6^ cells/animal/injected i.p.) were randomized into Group A, B, or C. **A** Three weeks post-last treatment, tumors were evaluated with IHC for CD3^+^ and CD8^+^ TILs (left panel) and % perforin-expressing CD3^+^ and CD8^+^ TILs in total TILs (middle panel). Representative images showing CD3^+^CD8^+^perforin^+^ T cells (right panel; white arrows). **B** Percentage of tumor-infiltrating CD3^+^CD4^+^Foxp3^+^ Treg cells in total CD4^+^ TILs (left panel), and the ratio of CD8^+^ TILs to tumor-infiltrating Treg cells (middle panel). Left panel, images showing CD3^+^CD4^+^Foxp3^+^ Treg cells (white arrows). **C** Quantification of FasL^+^CD31^+^ tumor endothelial cells (left panel). Middle panel, linear regression analysis indicated an inverse correlation between decreasing numbers of FasL^+^CD31^+^ tumor endothelial cells and increasing numbers of CD8^+^ TILs. Left panel, images showed FasL^+^CD31^+^ endothelial cells (yellow arrows) and CD8^+^TILs (white arrows). Data are representative of three separate experiments (*n* = 3 mice/group). One-way ANOVA and Tukey’s multiple comparisons test for significance were performed, and *, **, and *** denoted significant *P* < 0.05, *P* < 0.01, and *P* < 0.001, respectively. **D** Blood samples were collected a day before any treatment (baseline) and 3 weeks post the last treatment. Plasma was evaluated for Th1-related chemokines (CCL5, CCL4, CCL2, and CXCL9) in a cytokine bead array. Boxplots compared chemokine concentrations at baseline and 3 weeks post-last treatment (paired Student’s *t* test; * and ** denoted significant *P* < 0.05 and <0.001, respectively). **E** Post-treatment total Th1-related plasma chemokines and CXCL9 were presented as mean ± SEM. One-way ANOVA and Tukey’s multiple comparisons test for significance were performed. **F** Linear regression analysis indicated an inverse correlation between increasing numbers of CD3^+^ TILs (*r*^2^ = 0.690*; P* = 0.002) (left panel) and CD8^+^ TILs (*r*^2^ = 0.562*; P* = 0.008) (right panel) with increasing plasma CXCL9. Plasma chemokine data were representative of three separate experiments (*n* = 11–12 mice/group).

As T cells help controlling OC, we hypothesized that a larger number of TILs would be present in Group C than in other groups as the former showed the lowest tumor burden and longest OS. Seventy days post-tumor implantation, Groups A, B, C, and PBS-treated animals were sacrificed and tumors (if any) excised for immunohistochemistry (IHC) analysis (Supplementary Fig. 4 showing single color and merged images). We demonstrated significantly higher numbers of CD3^+^ (*P* = 0.039, *P* = 0.006, and *P* = 0.025 as compared to Groups A, B, and PBS-treatment, respectively) and CD8^+^ (*P* = 0.005, *P* = 0.023, and *P* = 0.003 as compared to Groups A, B, and PBS-treatment, respectively) TILs in Group C (Fig. 4A, right panel). We also detected significantly higher percentages of perforin-expressing CD3^+^ (*P* = 0.011, *P* = 0.034, and *P* = 0.023 as compared to Group A, B, and PBS-treatment, respectively) and CD8^+^ (*P* = <0.0001, *P* = 0.0002, and *P* = 0.0002 as compared to Groups A, B, and PBS-treatment, respectively) TILs in total CD3^+^ and CD8^+^ TILs, respectively, in Group C (Fig. 4A, left panel. IHC images indicating CD3^+^CD8^+^perforin^+^ T cells with white arrows). By comparing to Groups A (*P* = 0.050), B (*P* = 0.049), and PBS-treatment (*P* = 0.006), Group C demonstrated the lowest percentage of tumor-infiltrating CD3^+^CD4^+^Foxp3^+^ Treg cells in total CD4^+^ TILs (Fig. 4B, right panel. IHC images indicating CD3^+^CD4^+^Foxp3^+^ Treg cells with white arrows). Group C also showed the highest ratio of CD8^+^ TILs to tumor-infiltrating Treg cells (all *P* < 0.0001 when compared to Groups A, B, and PBS-treatment), suggesting a more favorable TME for effector T-cell responses than in other groups (Fig. 4B, left panel). Next, we hypothesized that FasL expressions were attenuated in Groups B and C animals that received ASA and anti-VEGF antibody as we had demonstrated in a previous study^19^. Indeed, we observed significantly less numbers of FasL^+^CD31^+^ tumor endothelial cells in Groups B and C when compared to Group A (vs. Group B: *P* = 0.031; vs. Group C: *P* = 0.008) and PBS-treatment (vs. Group B: *P* = 0.006; vs. Group C: *P* = 0.002) (Fig. 4C, left panel). We also observed an inverse correlation between reducing numbers of FasL^+^CD31^+^ endothelial cells and increasing numbers of CD8^+^ TILs in agreement with our previous study^19^ (*r*^2^ = 0.454; *P* = 0.033; Fig. 4C, right panel. IHC images indicating FasL^+^CD31^+^ endothelial cells with yellow arrows, and CD8^+^ TILs with white arrows). Thus, adding both ASA and low-dose IL-2 to the OCDC-Bev-Cy combinatorial regimen significantly increased total and perforin-expressing CD3^+^ and CD8^+^ TILs infiltrations while reducing tumor-infiltrating Treg cells. As with our previous study, the inclusion of ASA and anti-VEGF antibody significantly reduced tumor endothelial FasL expression that in turn was positively correlated with increased CD8^+^ TILs infiltration.

Next, we evaluated the plasma chemokine profiles and demonstrated a significant increase in post-treatment CCL5 (RANTES), CCL4 (MIP-1β), CCL2 (MCP-1), and CXCL9 (MIG) (Fig. 4D and Supplementary Fig. S5A, paired Student’s *t* tests). In particular, Group C demonstrated a significant increase in all of these chemokines when compared to Groups A and B (Fig. 4D). Moreover, Group C treatment induced the highest total amount of plasma Th1-polarizing chemokines and CXCL9 when compared to Groups A, B, and PBS-treatment (Fig. 4E). We demonstrated a significant positive correlation between an increased level of plasma CXCL9 and the number of tumor-infiltrating CD3^+^ and CD8^+^ T cells (Fig. 4F). No positive correlations were observed for the other chemokines (Supplementary Fig. S5B). Thus, the addition of both ASA and low-dose IL-2 to the OCDC-Bev-Cy combinatorial regimen induced a Th1-polarizing sera chemokine profile, particularly CXCL9 that was conducive for the recruitment of CD3^+^ and CD8^+^ TILs for tumor control.

### Adding ASA and low-dose IL-2 to an OCDC-Bev-Cy combinatorial regimen enhances tumor neoantigen-specific T-cell responses

**Fig. 5:**
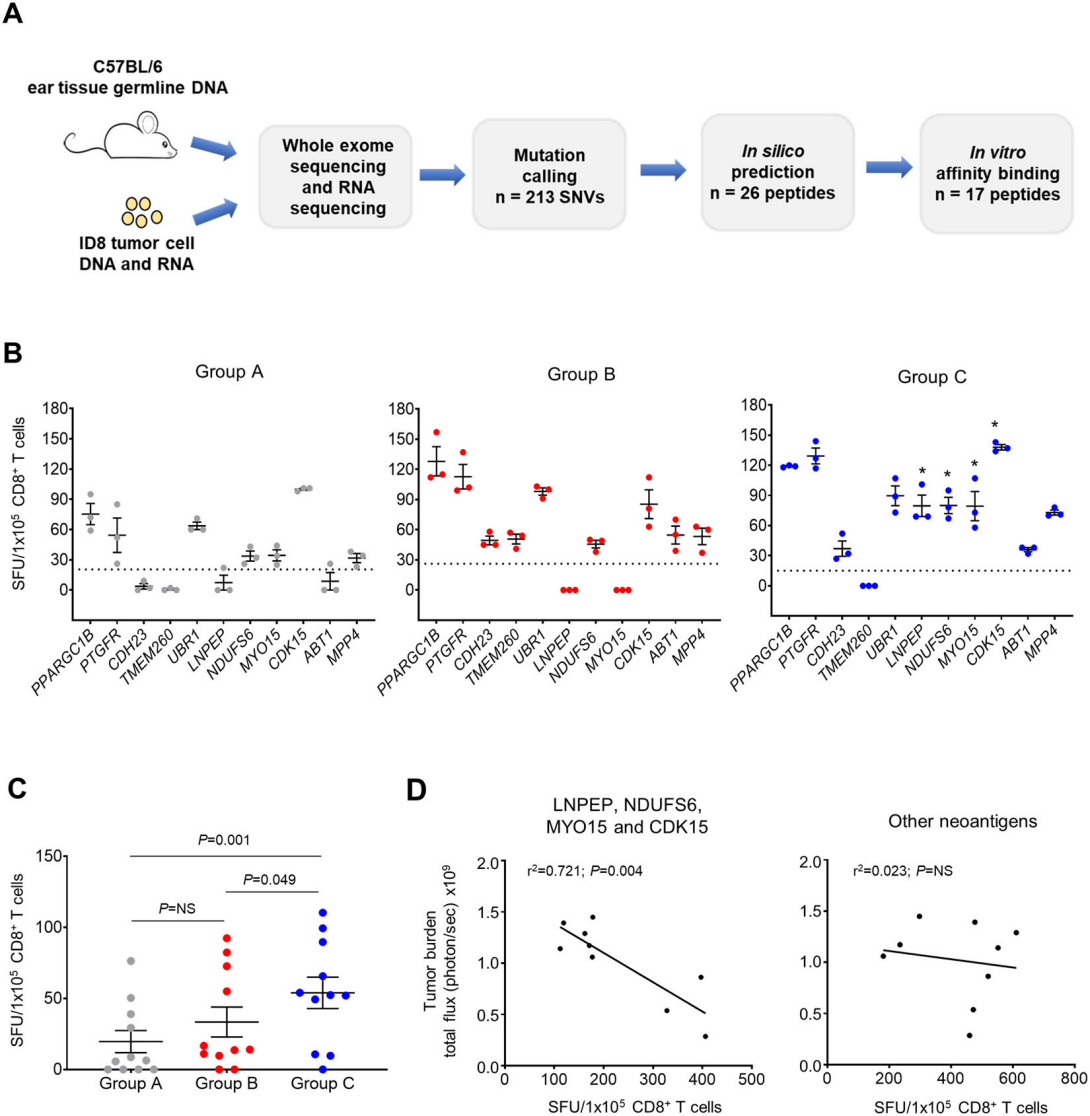
Adding ASA and low-dose IL-2 to OCDC-Bev-Cy combinatorial regimen enhances tumor neoantigen-specific T-cell responses. **A** Identification of MHC Class I-restricted neoantigens in ID8 ovarian tumor line. Seventeen of 216 single-nucleotide variants (SNVs) identified were considered immunogenic and tested in the IFN-γ ELISpot. **B** Three weeks post-last treatment, splenocytes were cultured with a pool of 17 neoantigen peptides (1 µg/ml per peptide) identified in (**A**). After 7 days, purified CD8^+^ T cells were evaluated in ELISpot for their ability to recognize individual neoantigen in the pool. In each experiment, three animals in the same group were pooled to obtain sufficient CD8^+^ T cells. Each dot represented the number of IFN-γ secreting spots/1 × 10^5^ CD8^+^ T cells of a pooled sample recognizing a neoantigen peptide. The results were subtracted from its respective unstimulated CD8^+^ T cells alone control. IFN-γ response was considered positive if it was above the threshold line based on its respective irrelevant melanoma TRP-2 peptide as a negative control (calculated as mean ± 3 × standard deviation). The results were reported as SFU/1 × 10^5^ CD8^+^ T cells. One-way ANOVA and Tukey’s multiple comparisons test for significance were performed, and * denoted *P* < 0.05 was significant. **C** Graph showed the mean number of IFN-γ-secreting neoantigen-specific CD8^+^ T cells elicited against the 11 neoantigens that showed reactivity in (**B**). Each dot represented IFN-γ response from a pooled CD8^+^ T-cell sample responding to a neoantigen. Results were reported as mean ± SEM. **D** Right panel showed linear regression analysis indicating a positive correlation between tumor burden (bioluminescence total flux (photon per second)× 10^9^) and the total number of IFN-γ spots elicited by neoantigens LNPEP, NDUFS6, MYO15, and CDK15 (*r*^2^ = 0.721; *P* = 0.004). Left panel, no positive correlation was observed for the rest of the neoantigens (*r*^2^ = 0.023; *P* = 0.695). The data were representative of three separate experiments with *n* = 9 animals/group. *P* < 0.05 was considered significant, while *P* > 0.05 was not significant (NS).

We previously demonstrated that OC patients receiving the baseline treatment elicited tumor neoantigen-specific T cells^6^, and we could further validate this finding in a patient in this study (Fig. 2D, E). As limited patient samples were available, we sought to evaluate neoantigen-specific T-cell responses in the ID8 model. We hypothesized that the addition of ASA and low-dose IL-2 to the baseline treatment could further enhance neoantigen recognition by T cells. Using ID8 tumor cells, we identified 213 somatic nonsynonymous mutations and of which 17 neoantigens were selected following in silico and in vitro validations (Fig. 5A and Supplementary Table S2). We focused on MHC Class I-restricted epitopes in the mice as well-established tools for in silico and in vitro validations were available as compared to those for MHC Class II-restricted epitopes. After a 7 day in vitro expansion of mouse splenocytes, we detected CD8^+^ T-cell IFN-γ responses to 11 of these neoantigens across all the regimens (Fig. 5B). Group C animals treated with additional ASA and low-dose IL-2 recognized 10 of these 11 neoantigens and elicited a significantly higher number of IFN-γ-secreting T cells against LNPEP, NDUFS6, MYO15, and CDK15 when compared to Groups A and B animals (*P* < 0.05) (Fig. 5B, third panel from left, indicated with asterisks). Overall, Group C elicited the highest number of IFN-γ-secreting neoantigen-specific T cells, while Group B demonstrated no significant difference with the baseline treatment group (Fig. 5C). By evaluating the number of neoantigen-specific CD8^+^ T cells elicited by each individual neoantigen and tumor burden measured 70 days post-tumor implantation (3 weeks post-last treatment) in the animals, we demonstrated a positive correlation between an increasing number of IFN-γ-secreting CD8^+^ T cells recognizing LNPEP, NDUFS6, MYO15, and CDK15 (individually and as a sum) and decreasing tumor burden. On the other hand, CD8^+^ T cells recognizing other tumor neoantigens did not show such correlation (Fig. 5D and Supplementary Fig. S5). Lastly, we did not observe any significant changes in the peripheral Treg cell population in all the groups (Supplementary Fig. S6; A showing gating strategy and B % peripheral Treg cells). Taken together, the addition of both ASA and low-dose IL-2 to the baseline treatment elicited more IFN-γ-secreting tumor neoantigen-specific T cells. This combinatorial approach elicited T cells against specific neoantigens (LNPEP, NDUFS6, MYO15, and CDK15) that were positively correlated with reduced tumor burden in the treated animals.

## Discussion

A combinatorial therapeutic strategy in OC that targets not only the tumor but also its TME is strongly warranted. Here, we presented evidence that the combinatorial use of ASA, low-dose IL-2, OCDC, Bev, and Cy (Cohort C regimen) increased the magnitude as well as the polyfunctional profile of OCDC vaccine-specific CD4^+^ and CD8^+^ T cells that were positively correlated with increased time-to-progression and 3-years OS in OC patients. Specific polyfunctional CD4^+^ and CD8^+^ T cells characterized by perforin, granzyme B, IFN-γ, and/or TNF-α expressions were elicited only in Cohort C patients. A higher 3-year OS rate was also observed in Cohort C (80%) than in Cohorts A and B receiving OCDC-Bev-Cy (40%) and ASA-OCDC-Bev-Cy regimens (40%), respectively (Cohort A data not shown).

Analysis of the ID8 OC model revealed a similar trend as in the OC patients whereby animals receiving ASA, low-dose IL-2, OCDC, anti-VEGF antibody (Bev), and Cy (Group C) demonstrated prolonged survival and reduced tumor burden. Tumor control by T cells was evident in these animals as significant infiltrations of CD3^+^ and CD8^+^ TILs including perforin-expression TILs were observed. Increased CD8^+^ TILs could be explained in part with ASA + anti-VEGF antibody treatment; in agreement with our previous study^19^, tumor endothelial FasL expression was reduced in Groups B and C animals receiving these two agents, and a positive correlation with increased CD8^+^ TILs was also observed. Similar to advanced human OCs, we found a high percentage of tumor-infiltrating Treg cells (~40% of total CD4^+^ TILs) in untreated (PBS) animals. As Treg cells could dampen the functions and tumor infiltration of effector T cells, we incorporated Cy in the regimens aiming to deplete Treg cells^32,33^. We observed moderate to marked reductions of tumor-infiltrating Treg cells in Groups A, B, and C that received Cy when compared to the PBS-treated group. Cy could provide additional benefits as it was shown to synergize with anti-VEGF antibody through so-called vascular normalization for Cy to better access tumor sites^34,35^ to disrupt tumor vasculature and exert anti-angiogenesis effects^36^.

Prolonged survival in Group C, as compared to Groups A and B, could be explained in part by the use of low-dose IL-2. It is reasonable to postulate that IL-2, a major T-cell growth factor, assists in the function and proliferation of OCDC vaccine-primed T cells. We observed improved vaccine-primed T-cell functions as demonstrated by increased polyfunctionality in Cohort C patients and increased neoantigen recognition and IFN-γ production in Group C animals. We also detected the highest numbers of CD3^+^ and CD8^+^ TILs, of which up to 40% was associated with a cytolytic functional profile, in Group C. Coupled with a low number of tumor-infiltrating Treg cells, Group C displayed a more favorable OC TME for tumor control than other groups as demonstrated by a significantly higher ratio of CD8^+^ TILs to tumor-infiltrating Treg cells. We also postulated that low-dose IL-2 augmented IFN-γ production in Group C animals, which the latter in turn induced CXCL9 which is an important chemoattractant of activated natural killer and Th1 cells into inflammation sites^37^ and for anti-angiogenesis^38^. Indeed, IFN-γ produced by vaccine-primed T cells and plasma CXCL9 was significantly increased in Group C. We also demonstrated that increasing plasma CXCL9 was positively correlated with increasing CD3^+^ and CD8^+^ TILs. Optimal ex vivo preparation of DC vaccines could also assist in eliciting beneficial antitumor as demonstrated by Kalinski et al.^39,40^. They demonstrated that type 1-polarized DCs (DC1s) matured ex vivo in the presence of IFNs and toll-like receptor agonists were more effective than “standard” DCs that were matured ex vivo in the presence of PGE2; while both types of DCs were effective in inducing CD8^+^ T-cell expansion, DC1s in particular enabled primed T cells to acquire cytolytic function and peripheral-type chemokine receptors such as CCR5 and CXCR3 (receptor for CXCL9)^39^. Indeed, the OCDC vaccine was prepared by using IFN-γ and lipopolysaccharides (LPS; TLR 4 agonist) as maturation stimuli and previously shown to produced a high level of IL-12p70 for priming T cells^5^. Activating DCs with other type 1-polarizing stimuli would be an interesting area to pursue.

Regarding ASA, a recent ASPREE trial demonstrated that healthy elderly individuals (≥70 years old) who used ASA daily for at least 4 years experienced increased cancer deaths^41^. More studies are needed to address the effects of ASA in elderly individuals. Such information would be useful in designing future OC trials involving ASA use in elderly OC patients. IL-2 is useful for expanding effector T cells in cancer therapy, however, it is also found to expand Treg cells in low-^42^ and high dose^43–45^. We did not observe an increased expansion of peripheral blood Treg cells in patients nor in animals receiving low-dose IL-2. Further studies are warranted to address this dual effect of IL-2 in OC cancer therapy.

Similar to our previous study^6^, we demonstrated that neoantigen-specific T cells were elicited in an OC patient and in the ID8 model. Recently, Martin et al. reported that an ID8-G7 tumor model (modified to express ovalbumin) failed to give rise to any neoantigen that facilitated long-term survival in the immunized animals^46^. The authors identified 92 single-nucleotide variants (SNVs) of which 39 were transcribed missense mutations. They showed that 7 of the top 17 predicted MHC class I binding neoantigens induced robust CD4^+^ and/or CD8^+^ T-cell response but none prolonged the survival of animals in prophylactic and therapeutic settings. We used the wild-type ID8 cell line (no ovalbumin expression) and identified 216 SNVs, of which 17 were immunogenic following in silico and in vitro validations. We demonstrated that 11 of these neoantigens induced IFN-γ responses, and 4 of them were associated with reduced tumor burden in Group C. Differences in our results could be explained by the fact that we and Martin et al. discovered different sets of neoantigens through the use of different algorithms for neoantigen predictions; we developed an in-house algorithm that adapted Burrows–Wheeler Aligner (BWA) and Genome Analysis Toolkit (GATK) to analyze murine genome sequences, whereas Martin el al. utilized the Structure Motif Analysis tool (SMAtool) for their neoepitope predictions. We also used different ID8 cell lines (wild-type ID8 vs. ID8-G7) for whole-exome and RNA sequencing. Although we detected more SNVs (216 vs. 92), we and Martin et al. found only 17 immunogenic neoantigens following in vitro MHC binding in agreement that OC is an intermediate/low mutational burden tumor. None of the neoantigens (LNPEP, NDUFS6, MYO15, and CDK15) we found that was associated with reduced tumor burden was identified by Martin et al. Interestingly, we and Martin et al. identified a neoantigen from Tle1 gene that failed to elicit T-cell responses in the animals. Our results demonstrated that targeting neoantigens is beneficial in OC. This also highlighted a strong need to optimize and standardize neoantigen prediction algorithms to improve their accuracy for long-term translational use.

As the single therapy approach has so far shown minimal efficacy in OC, a combinatorial approach is highly warranted. We demonstrated that the combinatorial use of ASA, low-dose IL-2, OCDC, Bev, and Cy was efficacious in OC. We provided evidence that adding ASA and IL-2 to the OCDC-Bev-Cy combinatorial regimen could modulate the OC TME to augment OCDC vaccine-primed T-cell responses. We detected increased cytolytic TILs and reduced tumor-infiltrating Treg cells, favoring a Th1-polarizing TME. Reduced tumor endothelial FasL expression and increased plasma CXCL9 were positively correlated with increased TILs, further augmenting Th1-polarizing responses. Through this co-clinical small-scale study, we also demonstrated that the syngeneic ID8 tumor model could serve as a useful tool for predicting different therapeutic strategies in OC for future clinical trial designs.

## Methods

### Clinical trial design and patient cohorts

Patients with recurrent ovarian, fallopian tube, or primary peritoneal cancer were enrolled in phase I, a single-center study comprised of various sequential cohorts (NCT01132014). The primary objective was to establish the safety and biological activity of OCDC, a vaccine prepared from autologous DCs pulsed with oxidized autologous whole-tumor lysate, in combination with Bev, Cy, ASA, and/or low-dose IL-2 (Fig. 1A). OCDC preparation was previously described^5,6^. Thirty patients were enrolled (ten patients per cohort). Cohort A received intravenous Cy (200 mg/m^2^) followed by OCDC (5–10 × 10^6^ DCs/dose given intranodally every 3 weeks, five doses in total) plus intravenous Bev (anti-VEGF blocking antibody, 15 mg/kg, given on the same day as OCDC) on the next day every 3 weeks. Cohort B received the above regimen plus oral ASA (325 mg of enteric-coated aspirin from the day of first OCDC to day 84 or as tolerated). Subcutaneous low-dose IL-2 (2MIU/dose given for 5 consecutive days following each OCDC) was given to Cohort C in addition to the regimen described for Cohort B. Twelve to 15 weeks post-OCDC vaccination, patients with no evidence of disease or stable disease were offered a maintenance regimen. Patients with progressive diseases went off the study. Additional inclusion criteria required that patients were ≥18 years old, had sufficient tumor previously harvested at secondary cytoreductive surgery for lysate preparation, had completed physician’s choice chemotherapy after secondary debulking and had a baseline Eastern Cooperative Oncology Group (ECOG) performance status 0–1. After enrolment, patients underwent 10–15 liters apheresis to harvest elutriated monocytes for OCDC production. All the patients were followed up for 3 years. The study was approved by the US FDA (BB-IND-14269) and by the University of Pennsylvania’s Institutional Review Board. All patients gave written informed consent before initiation of any study procedures.

### Patient OCDC vaccine manufacturing, vaccination, and monitoring

OCDC vaccines were generated at the Clinical Cell and Vaccine Production Facility (CVPF) at the University of Pennsylvania as previously described^5,6^. Following 10–15 liters of apheresis, the patient’s PBMCs were elutriated (fraction 5) and the monocytes cultured in CellGenix DC media (CellGenix, Germany), 2% human pooled AB serum (Valley Biomedical Inc., Winchester, USA), L-glutamine (2 mM), penicillin (100 U/ml), streptomycin (100 μg/ml; Cellgro), clinical-grade human GM-CSF (500 IU/ml; Sargramostim, Bayer Healthcare Pharmaceuticals), and animal-free research-grade IL-4 (250 IU/ml; R&D Systems). Day 4, output cells were evaluated for DC markers CD11c (clone B-ly6), CD14 (clone M5E2), and HLA-DR (clone L243) (all from BD Biosciences) and were found to be >70% pure. Following overnight pulsing with hypochlorous acid (HOCl)-oxidized autologous whole-tumor lysate, the DCs were activated with LPS (60 EU/ml; *Escherichia coli* O:113; a gift from A. Suffredini at the National Institutes of Health (NIH)] and recombinant human IFN-γ (2000 IU/ml; InterMune, California, USA). OCDC vaccines met the release criteria in all the patients. Vaccine aliquots (~5–10 × 10^6^ DCs per dose) were cryopreserved at −140 °C, thawed, and washed before each administration. Patients received five doses of OCDC vaccines intranodally every 3 weeks under ultrasound guidance and continued on a monthly maintenance regimen until disease progression or exhaustion of vaccine supply. Safety was determined using the National Cancer Institute Common Terminology Criteria for Adverse Events version 4.0. Patients underwent a CT scan at enrolment and on day 114 (EOS). Clinical response was based on the Response Evaluation Criteria in Solid Tumors (RECIST) 1.1 and immune-related response criteria. Patients continued with either maintenance vaccination plus Bev and Cy ± ASA ± low-dose IL-2, or went off the study. Blood samples were collected from all patients prior to treatment for baseline immune assessment (pre-treatment; pre-VAX), during the study, and at EOS (post-treatment). All patients’ samples were transferred and analyzed at the Center of Experimental Therapeutics (CTE) at the Lausanne Branch of the Ludwig Institute for Cancer Research.

### IFN-γ ELISpot of human OCDC vaccine-specific and neoantigen T cells

We successfully collected PBMCs from 12 of 20 patients enrolled in Cohorts B and C (6 from each cohort) at various time points specified in Fig. 1A for analysis. To evaluate IFN-γ-secreting OCDC vaccine-specific T cells before (pre-vax; day −1), during (day 20 and 62) and after (EOS) treatment in these patients, cryopreserved PBMCs were thawed, rested overnight in Roswell Park Memorial Institute (RPMI) 1640 media containing 10% fetal bovine serum (FBS), penicillin (100 U/ml) and streptomycin (100 μg/ml) at 37 °C, 5% CO_2_ (all from Invitrogen, CA, USA). The next day, the OCDC vaccine was thawed, washed, and immediately added to PBMCs at 1 ODCD:10 PBMCs ratio in pre-coated 96-well Enzyme-Linked ImmunoSpot (ELISpot) plates (Mabtech AB, France) and evaluated. For IFN-γ-secreting neoantigen-specific T-cell analysis in patient B-08, cryopreserved PBMCs from EOS were thawed, washed, and stimulated with neoepitope (STELMRRVSRFQIAQ) or wild-type (STELMRRVRRFQIAQ) cognate peptide (1 µg/ml per peptide) in RPMI media containing penicillin (100 U/ml), streptomycin (100 μg/ml), 2-mercaptoethanol (50 µM), and 8% AB pooled human serum (Biowest, France). Recombinant human IL-2 (100 IU/ml, Proleukin^®^, Bayer HealthCare Pharmaceuticals, CA, USA) was added after 48 h. Day 12, IFN-γ ELISpot assays were performed. PBMCs stimulated with Staphylococcal Enterotoxin (SEB) served as positive controls, while PBMCs stimulated with media were used as background controls. In total, 2 × 10^5^ PBMC were plated in duplicates or triplicate for each condition. After 16–18 h of stimulation, the plates were washed according to the manufacturer’s instruction and counted with AID-Spot Robot ELISpot reader (AutoImmun Diagnostika GMBH, Germany). Results were expressed as spots-forming units (SFU) per 1 × 10^6^ PBMCs.

### Flow cytometry analysis of human OCDC vaccine-specific and neoantigen T cells

To determine the phenotype and cytokine profiles of OCDC vaccine-specific CD4^+^ and CD8^+^ T cells by flow cytometry, patients’ cryopreserved PBMCs were thawed and rested overnight in RPMI media containing 10% FBS, penicillin (100 U/ml) and streptomycin (100 μg/ml) at 37 °C, 5% CO_2_. The next day, autologous OCDC vaccine was thawed, washed with RPMI, 10% FBS, and immediately added to the PBMCs at 1 OCDC:10 PBMCs ratio in the presence of brefeldin A (1 µg; BD Biosciences, San Jose, USA) and for 16–18 h incubation at 37 °C, 5% CO_2_. Then, the cells were washed with PBS and stained for 20 min on ice with anti-CD3 (clone SK7; Biolegend), anti-CD8 (clone RPA-T8; Biolegend), anti-CD4 (clone RPA-T4; BD Biosciences), anti-CD137 (4-1BB, clone 4B4-1; Biolegend), and a viability dye (Zombie UV, Biolegend). After fixation and permeabilization step (BD Cytofix/Cytoperm Kit) for 20 min at 4 °C, cells were intracellularly stained with anti-Granzyme B (clone GB11; Invitrogen, CA, USA), anti-Perforin (clone B-D48; Biolegend), anti-IL-2 (clone MQ1-17H12; BD Biosciences), anti-TNF-α (clone MAb11; BD Biosciences), and anti-IFN-γ (clone B27; BD Biosciences) for 20 min on ice. In another staining panel, cells were stained intracellularly with anti-IL-17 (clone BL168; Biolegend) and anti-Ki-67 (clone B-ly6; BD Biosciences). To determine exhaustion markers, the cells were stained for 20 min on ice with anti-CD3 (Biolegend), anti-CD8 (Biolegend), anti-CD4 (BD Biosciences), anti-PD1 (clone EH12.2H7; Biolegend), anti-TIM3 (clone 344823; R&D Systems, MN, USA), anti-LAG-3 (clone 17B4; Enzo Life Sciences (ELS) AG, Switzerland), anti-TIGIT (clone 741182, R&D Systems), anti-2B4/CD244 (clone C1.7; Biolegend) and a viability dye (Zombie UV, Biolegend). After fixation and permeabilization treatment as described above, cells were intracellularly stained with anti-CTLA-4 (clone BNI3; BD Biosciences) for 20 min on ice.

To determine intracellular TNF-α and IFN-γ profile of neoantigen-specific CD4^+^ and CD8^+^ T cells in patient B-08, cryopreserved PBMCs were thawed, washed, and stimulated the cells for 16–18 h with neoantigen peptide pool (final concentration of 1 µg/ml per peptide; Supplementary Table S1) using same the culture condition as described for this patient’s ELISpot analysis. PBMCs stimulated with media alone were used as a background control, while PBMCs stimulated with anti-CD3 antibody (final concentration of 1 µg/ml, 4 °C overnight; clone OKT3, Mabtech) were used as a positive control. The cells were stained as described above.

To determine Treg cell markers in patients’ PBMCs at EOS, cells were stained for 20 min on ice with anti-CD8 (Biolegend), anti-CD3 (Biolegend), anti-CD4 (BD Biosciences), anti-CD25 (clone 2A3; BD Biosciences), anti-TIGIT (R&D Systems), anti-CD45A (clone HI100; BD Biosciences), anti-CD127 (clone R34.34; Beckman Coulter, France), anti-CD39 (clone A1; Biolegend), and viability dye Zombie UV (Biolegend). After fixation and permeabilization treatment (eBiosciences Foxp3 kit) for 30 min at 4 °C, cells were intracellularly stained with anti-Foxp3 (clone 236 A/E7; eBiosciences) and anti-Helios (clone 22F6, Biolegend). After the last staining step, all the samples were washed twice with PBS before acquiring on a 5-laser BD Fortessa instrument equipped with the FACS Diva software. The analysis was performed with FlowJo v10.5 (FLOWJO.LLC) and SPICE 5.1 software.

### Human neoepitope predictions

Whole-exome sequencing analyses for mutation calling and high-resolution HLA typing were performed as previously described^47^. MixMHCpred.v2.0.2 and MixMHC2pred.v1 was used to predict the binding of candidate peptides incorporating somatic nonsynonymous mutations and their wild-type counterparts (8–12 mer peptides to HLA class I alleles and 12–19 mer peptides to HLA Class II alleles)^48–50^. Predicted mutated peptides were prioritized based on the following parameters (by decreasing order of importance): predicted binding rank, the median expression of the gene in healthy ovarian tissues in GTEx^51^, the representation of the peptide in ipMSDB^52^. Peptides with binding rank >5%, gene expression in GTEx < 1TPM, and peptides with low representation in ipMSDB (<4 class I and class II peptides) were removed from the list. The peptides were grouped by source gene. The best peptides of each gene and for each HLA allele were selected for further analysis.

### Synthesis of human and mouse neoantigen peptides

Human candidate neoepitopes and their wild-type counterpart peptides were synthesized at >90% high-performance liquid chromatography (HPLC) purity. For mouse ID8, 10-mer candidate neoepitope peptides were synthesized at ≥79% HPLC purity. All peptides were synthesized at the Protein and Peptide Chemistry Facility (PPCF), University of Lausanne, Switzerland.

### Mouse cell line

The ID8 cell line, derived from spontaneous in vitro malignant transformation of C57BL/6 mouse ovarian surface epithelial cells (gift from Prof. Iain McNeish, University of Glasgow, UK), closely represents human high-grade serous OC in terms of disseminated peritoneal tumors and ascites fluid formation^53^. It was maintained in Dulbecco’s modified Eagle’s medium (DMEM) containing 4% FBS, penicillin (100 U/ml), streptomycin (100 µg/ml) (all from Invitrogen), and 1% insulin–transferrin–selenium (ITS-G; Gibco, Thermo Fisher Scientific, MA, USA) at 37 °C, 5% CO_2_. It was tested regularly for mycoplasma and found to be negative.

### Mouse OCDC vaccine preparation

Mouse OCDC vaccine preparation was previously described^5^. Briefly, bone marrow cells from mouse femurs and tibias were cultured at 1–2 × 10^6^ cells/ml in complete Iscove’s Modified Dulbecco’s Medium (IMDM) containing 10% FBS, 50 µM 2-mercaptoethanol, 100 U/ml penicillin, 100 µg/ml streptomycin (all from Invitrogen), and 1000 IU/ml recombinant mouse granulocyte–macrophage colony-stimulating factor (GM-CSF; PeproTech, UK). Day 3, floating cells representing granulocytes were removed, and fresh complete IMDM and 1000 IU/ml GM-CSF were added. Day 5, recombinant mouse IL-4 (100 IU/ml; PeproTech, UK) was added. Day 6, HOCl-oxidized ID8 tumor cell lysate were cocultured with DCs at 1:1 ratio for 20 h. DCs were stimulated with lipopolysaccharide (LPS) [120 EU/ml, *Escherichia Coli* O:113; InvivoGen Europe, France] and IFN-γ (4000 IU/ml; PeproTech, UK) for 16 h and used. HOCl oxidation of ID8 tumor lysate was previously described (ref. ^5^; main paper). Briefly, 60 µM HOCl solution was prepared by diluting the stock NaOCl reagent (Sigma-Aldrich Chemie GmbH) with PBS (Invitrogen) and adding immediately to ID8 cells to give 1 × 10^6^ cells/ml. ID8 cells were incubated for 1 h at 37 °C, 5% CO_2_ to induce oxidation-dependent cell death, and then subjected to six freeze–thaw cycles to complete cell fragmentation before coculturing with DCs.

### In vivo treatments in mice

Syngeneic 8–10-week-old female C57BL/6 mice (Envigo, France) were implanted with 5 × 10^6^ ID8 tumor cells intraperitoneally (i.p.) in 200 µl of sterile saline and randomized into Group A, B, or C to receive specific treatments (Fig. 3A). In all groups, tumor-bearing animals received Cy (40 mg/kg, prepared in sterile PBS and given i.p.; Sigma-Aldrich Chemie GmbH, Switzerland) a day before each OCDC and anti-VEGF antibody (2 mg/kg, clone B20-4.1.1 mouse IgG2a, prepared in sterile PBS and given i.p.; Absolute Antibody, UK) together with each OCDC. In Groups B and C, animals were given ASA via drinking water (100 mg/kg, assuming daily consumption of ~3 ml at 0.7 mg/ml prepared in sterile deionized water^54^; Sigma-Aldrich Chemie GmbH). In Group C, animals received recombinant mouse IL-2 (15 µg/kg, prepared in sterile PBS given i.p.; PeproTech, UK) on two consecutive days following OCDC vaccination. OCDC was given intradermally in the groin region (1 × 10^6^ DCs/50 µl sterile PBS/mouse). As controls, tumor-bearing animals were treated with PBS only following the Group C schedule. As treatment controls, tumor-bearing animals were treated with Cy, anti-VEGF antibody, ASA, low-dose IL-2 only, or Cy+anti-VEGF antibody following Group C schedule. Animals were weighed at least twice weekly and euthanized when their body weight exceeded >20% as a surrogate endpoint for survival or when they became distressed and moribund in compliance with the institutional animal care guidelines. For neoantigen T-cell responses, animals were sacrificed 3 weeks after the last treatment (70 days post-tumor implantation). Animal experimentation procedures were performed according to the animal protocols approved by the Veterinary Service of Canton Vaud, Switzerland.

### Qualification of ID8 tumor burden with bioluminescence imaging

ID8 tumor cells (5 × 10^6^/mouse) expressing luciferase were injected i.p. into 8–10-week-old C57/BL6 female mice as described earlier. Day 19 post-tumor implantation and before the first treatment, tumor burden was measured by bioluminescence quantification of luciferase activity (reported as photons/sec). Bioluminescence imaging (Xenogen IVIS lumina II, PerkinElmer, Switzerland) was performed once every 2 weeks thereafter before the onset of ascites formation in PBS-treated control mice.

### ID8 tumor somatic nonsynonymous mutations identification and validation

Genomic DNA and RNA from ID8 tumor cells and DNA from C57BL/7 mouse ear tissue were isolated using DNeasy or RNeasy kits (Qiagen AG, Switzerland) following the manufacturer’s instructions. Whole-exome sequencing was performed (Fasteris SA, Switzerland). Somatic nonsynonymous mutations in the ID8 tumor cell were identified using a pipeline by aligning the whole-exome sequences to the reference C57BL/6J mouse genome using Burrows–Wheeler Aligner (BWA), and performing variant-calling with Genome Analysis Toolkit (GATK) as previously^6^ but modified for murine genome sequences. The mutations were evaluated with in silico predictions with NetMHC (version 4.0) and NetMHCpan (version 3.0), and ranked according to their binding affinity to the mouse MHC class I H-2Db and H2Kb alleles. Gene expressions of the identified somatic mutations were determined with RNA sequencing. Additional filtering of the neoepitope candidates was performed according to their gene expression with RNA sequences alignment (STAR) and gene annotation (GENCODE). Finally, the top 2% of the neoepitopes that showed a higher binding affinity to MHC Class I (H2-Db and/or H2Kb) than their wild-type counterpart and were expressed in at least two out of the three triplicates were selected for further validation. Following that, in vitro ELISA assay was performed as previously described to determine the candidate neoepitope binding to MHC Class I H2-Db and H2Kb alleles^55^. Immunogenic neoepitopes were used in the mouse interferon (IFN)-γ ELISpot.

### Multiplex immunohistochemistry staining of ID8 OC tumors

Three weeks after the last treatment (70 days post-tumor implantation), mice were sacrificed and freshly dissected ID8 OC tumors were fixed in 4% paraformaldehyde solution for 24 h at room temperature. To prepare paraffin-embedded tissue blocks, the tumors were dehydrated through 70%, 80%, and 95% alcohol (45 min each), followed by three changes of 100% alcohol (1 h each), clearing through two changes of xylene (1 h each) and finally immersed in three changes of paraffin (1 h each). Sections of paraffin-embedded tissue blocks (5-µm thick) were prepared on a microtome and transferred onto glass slides for multiplex immunohistochemistry. The slides were dried overnight and stored at 4 °C until ready for use. Multiplex IHC assays were performed on a VENTANA DISCOVERY Ultra automated staining instrument (Roche Diagnostics, Indianapolis, USA), using VENTANA reagents except as noted, according to the manufacturer’s instructions and as previously described^56^. The following primary antibodies against mouse markers were used: (a) anti-CD3 (rat monoclonal, clone SP7, Abcam plc, UK); (b) anti-CD8 (rat monoclonal, clone 4SM15, Thermo Fisher Scientific, Switzerland); (c) anti-CD4 (rat monoclonal, clone 4SM95, Thermo Fisher Scientific); (d) anti-CD31 (rat monoclonal, clone SZ31, Dianova GmbH, Germany); (e) anti-perforin (rat monoclonal, clone CB5.4, Abcam plc); (f) anti-FOXP3 (rat monoclonal, clone FJK-16s, Thermo Fisher Scientific); (g) anti-WT1 (rabbit monoclonal, clone CAN-R9(IHC)-56-2, Abcam plc); and (h) anti-FasL (rabbit polyclonal, Bioss Antibodies, Massachusetts USA). The following secondary antibodies were used: (a) ImmPRESS^®^ horseradish peroxidase (HRP) goat anti-rat IgG (Vector Laboratories, CA, USA), (b) ImmPRESS^®^ HRP horse anti-rabbit IgG (Vector laboratories). The slides were mounted with Fluoromount-G™ mounting medium containing DAPI (Thermo Fisher Scientific) and analyzed within 48 h.

### Multispectral imaging and data analysis

Multiplex immunofluorescence (IF) images were acquired on the Vectra^®^ Polaris automated quantitative pathology imaging system (Akoya Biosciences, Marlborough, MA, USA), allowing the unmixing of spectrally overlapping fluorophores and tissue autofluorescence of whole slide scans. For the optimal IF signal unmixing (individual spectral peaks) and the subsequent multiplex analysis, a spectral library containing the individual emitting spectral peaks of all the five to six fluorophores were created and validated using the inForm Analysis software (Akoya Biosciences). The phenotyping analysis was performed using inForm 2.4.8 image analysis software (Akoya Biosciences) enabling a per-cell analysis of weak and/or spectrally overlapping IF markers of multiplex-stained tissue sections. The images were first segmented into specific tissue categories of the tumor, stroma, and no tissue, based on the WT1 (OC tumor antigen expressed on ID8 tumor cells) and DAPI staining using the inForm Tissue Finder™ algorithms. Then, individual cells were segmented using the counterstained-based adaptive cell-segmentation algorithm. Quantification of the immune cells was finally performed using the inForm active learning phenotyping algorithm by assigning the different cell phenotypes across several images representative of the whole scan. InForm software was trained to recognize cell phenotypes according to these three staining panels: (1) panel 1—CD8-, CD3-, WT1-, Perforin-positive cells; (2) panel 2—CD4-, CD3-, WT1-, Foxp3-positive cells; and (3) panel 3—CD8-, WT1-, FasL-, CD31-positive cells. This algorithm was then applied on the whole scan by batch to quantify all the different cell types and a home-made R-script is then used to retrieve all combined phenotype cells and scoring in an output excel file.

### Mouse peripheral blood Treg cell staining

About 50 µl of blood per draw was collected into Lithium heparin-coated Eppendorf tubes (Fisher Scientific AG, Switzerland) on ice and centrifuged at 1500 × *g* for 15 min at 4 °C. Red cells were removed with ammonium–chloride–potassium (ACK) lysis buffer (Sigma-Aldrich Chemie GmbH, Switzerland), washed twice with PBS and blocked with staining buffer (PBS containing anti-mouse CD16/CD32 at 1 µg/ml) for 10 min on ice. Then, cells were stained with anti-CD3 (clone 17A2, eBioscience), anti-CD4 (clone RM4-5, eBioscience) and anti-CD25 (clone PC61, eBioscience) for 30 min on ice. Following that, cells were subjected to fixation and permeabilization treatment (eBioscience) for 30 min at 4 °C and stained overnight with anti-Foxp3 (clone FJK-16s, eBioscience). The next day, cells were washed twice with staining buffer, collected on the same day, and interrogated using BD Canto flow cytometer (Becton Dickinson). Data were analyzed with Pro CellQuest software. T cells that were positive for CD3, CD4, CD25, and Foxp3 were expressed as a percentage of all the CD4 + T cells.

### Mouse plasma chemokine bead array analysis

To measure the plasma chemokines, about 50 µl of blood per draw was collected a day prior to any treatment (baseline) and 3 weeks after the last treatment (day 70 post-tumor implantation). The blood samples were processed as described above. The top phase that consisted of the plasma was carefully removed to avoid any leukocytes and red cells and stored at −80 °C until ready to use. The plasma chemokines were evaluated with the LEGENDplex™ Mouse Proinflammatory Chemokine Panel following the manufacturer’s protocol (Biolegend, San Diego, CA, USA).

### IFN-γ production by mouse neoantigen-specific T cells

Three weeks after the last treatment, mouse splenocytes were harvested and cultured with a pool of 17 neoantigen peptides (1 µg/ml per peptide) identified in the ID8 cell line (Supplementary Table S2). Splenocytes were cultured in complete RPMI media containing 10% FBS, 2-mercaptoethanol (50 µM), penicillin (100 U/ml), and streptomycin (100 µg/ml) and recombinant mouse IL-2 (300 IU/ml; PeproTech). Fresh IL-2 and complete RMPI media were replenished every 2–3 days. Day 8, CD8^+^ T cells in the cultures were isolated by negative selection (Miltenyi Biotec Swiss AG, Switzerland) and assessed in IFN-γ ELISpot assay for their ability to recognize specific neoantigen peptides. In each experiment, three mice in the same group were pooled to obtain sufficient CD8^+^ T cells. Irradiated splenocytes from normal mice served as antigen-presenting cells (APCs) and pre-pulsed with individual neoantigen peptide (1 µg/ml) before coculturing with CD8^+^ T cells at a ratio of 10 T cells:1 APC for 16 h at 37 °C. CD8^+^ T cells exposed to phorbol 12‐myristate 13‐acetate (PMA) and ionomycin (both from Sigma-Aldrich Chemie GmbH) served as positive controls. CD8^+^ T cells alone served as background and T cells cocultured with irradiated APCs pulsed with melanoma tyrosinase-related protein (TRP)-2 served as irrelevant peptide controls. ELISpot was performed following the manufacturer’s protocol (Mabtech AB). The number of IFN-γ-producing cells was counted with AID-Spot Robot ELISpot reader (AutoImmun Diagnostika GMBH) and expressed as SFU per 1 × 10^5^ mouse CD8^+^ T cells. After subtracting the count from its respective background, a positive neoantigen peptide response was determined as having more spots than its respective irrelevant TRP-2 peptide control + 3× standard deviation (SD).

### Statistical analysis

Median survival times were computed using Kaplan–Meier methods and the log-rank test. The analysis of variance (ANOVA) was used to compare more than two groups. When the ANOVA was significant, Tukey’s multiple comparison test was used to conduct pairwise comparisons unless otherwise stated. A two-tailed Student’s *t* test (paired and unpaired) was used to compare means of continuous measurements between two groups. Differences were considered statistically significant when *P* < 0.05. GraphPad Prism 8 (San Diego, CA, USA) was used. Flow cytometry data were analyzed with FlowJo v10.5 (FlowJo, LLC) and SPICE 5.1 software^57^. *P* values for SPICE pies were determined with Wilcoxon rank-sum test.

### Reporting summary

Further information on research design is available in the Nature Research Reporting Summary linked to this article.

## Supplementary information

Reporting Summary

Supplementary Information

## Data Availability

The datasets generated and/or analyzed during this study are available from the corresponding authors.
