# Author Correction: Personalized cancer vaccine strategy elicits polyfunctional T cells and demonstrates clinical benefits in ovarian cancer

**DOI:** 10.1038/s41541-021-00332-5

**Published:** 2021-05-04

**Authors:** Janos L. Tanyi, Cheryl L.-L. Chiang, Johanna Chiffelle, Anne-Christine Thierry, Petra Baumgartener, Florian Huber, Christine Goepfert, David Tarussio, Stephanie Tissot, Drew A. Torigian, Harvey L. Nisenbaum, Brian J. Stevenson, Hajer Fritah Guiren, Ritaparna Ahmed, Anne-Laure Huguenin-Bergenat, Emese Zsiros, Michal Bassani-Sternberg, Rosemarie Mick, Daniel J. Powell, George Coukos, Alexandre Harari, Lana E. Kandalaft

**Affiliations:** 1 Ovarian Cancer Research Center, Abramson Cancer Center, Perelman School of Medicine, University of Pennsylvania, Philadelphia, PA USA; 2 Department of Oncology, Lausanne University Hospital (CHUV), Ludwig Institute for Cancer Research, University of Lausanne, Lausanne, Switzerland; 3 Center of Experimental Therapeutics, Department of Oncology, Lausanne University Hospital (CHUV), Lausanne, Switzerland; 4 Institute of Animal Pathology, COMPATH, Vetsuisse Faculty, University of Bern, Bern, Switzerland; 5 School of Life Sciences, Ecole Polytechnique Fédérale de Lausanne, Lausanne, Switzerland; 6 Department of Radiology, Hospital of the University of Pennsylvania, Philadelphia, PA USA; 7 Department of Biostatistics and Epidemiology, Perelman School of Medicine, University of Pennsylvania, Philadelphia, PA USA

**Keywords:** Cancer, Immunology, Cancer immunotherapy, Cancer therapy, Tumour immunology

Correction to: *npj Vaccines* 10.1038/s41541-021-00297-5, published online 15 March 2021

The original version of the published Article contained an error in the Figure legend of Figure 4. This has been corrected in the HTML and PDF version of the Article.

