# Supplementary material for: Personalized cancer vaccine strategy elicits polyfunctional T cells and demonstrates clinical benefits in ovarian cancer
Source: NPJ Vaccines. 2021 Mar 15;6:36. doi: 10.1038/s41541-021-00297-5 (PMC7960755; doi:10.1038/s41541-021-00297-5)
Supplement: Supplementary file 1 — Reporting Summary [file 41541_2021_297_MOESM1_ESM.pdf]

nature research

Corresponding author(s): Cheryl Lai-Lai Cheryl

Last updated by author(s): 09.02.2021

Reporting Summary

Nature Research wishes to improve the reproducibility of the work that we publish. This form provides structure for consistency and transparency in reporting. For further information on Nature Research policies, see our Editorial Policies and the Editorial Policy Checklist.

Statistics

For all statistical analyses, confirm that the following items are present in the figure legend, table legend, main text, or Methods section.

n/a	Confirmed	
☐	☒	The exact sample size (*n*) for each experimental group/condition, given as a discrete number and unit of measurement
☐	☒	A statement on whether measurements were taken from distinct samples or whether the same sample was measured repeatedly
☐	☒	The statistical test(s) used AND whether they are one- or two-sided<br>*Only common tests should be described solely by name; describe more complex techniques in the Methods section.*
☐	☒	A description of all covariates tested
☐	☒	A description of any assumptions or corrections, such as tests of normality and adjustment for multiple comparisons
☐	☒	A full description of the statistical parameters including central tendency (e.g. means) or other basic estimates (e.g. regression coefficient) AND variation (e.g. standard deviation) or associated estimates of uncertainty (e.g. confidence intervals)
☐	☒	For null hypothesis testing, the test statistic (e.g. *F*, *t*, *r*) with confidence intervals, effect sizes, degrees of freedom and *P* value noted<br>*Give P values as exact values whenever suitable.*
☒	☐	For Bayesian analysis, information on the choice of priors and Markov chain Monte Carlo settings
☒	☐	For hierarchical and complex designs, identification of the appropriate level for tests and full reporting of outcomes
☒	☐	Estimates of effect sizes (e.g. Cohen's *d*, Pearson's *r*), indicating how they were calculated

Our web collection on statistics for biologists contains articles on many of the points above.

Software and code

Policy information about availability of computer code

Data collection	No software was used.
Data analysis	MixMHCpred.v2.0.2, MixMHC2pred.v1, ipMSDB, NetMHC (version4.0), NetMHCpan (version 3.0), and GENOCODE

For manuscripts utilizing custom algorithms or software that are central to the research but not yet described in published literature, software must be made available to editors and reviewers. We strongly encourage code deposition in a community repository (e.g. GitHub). See the Nature Research guidelines for submitting code & software for further information.

Data

Policy information about availability of data

All manuscripts must include a data availability statement. This statement should provide the following information, where applicable:

- Accession codes, unique identifiers, or web links for publicly available datasets
- A list of figures that have associated raw data
- A description of any restrictions on data availability

The data sets generated and/or analyzed during the current study are available from the corresponding author.

Field-specific reporting

Please select the one below that is the best fit for your research. If you are not sure, read the appropriate sections before making your selection.

☒ Life sciences ☐ Behavioural & social sciences ☐ Ecological, evolutionary & environmental sciences

For a reference copy of the document with all sections, see nature.com/documents/nr-reporting-summary-flat.pdf

Life sciences study design

All studies must disclose on these points even when the disclosure is negative.

Sample size	Sample size was determined with pilot experiments for median results and standard error of the mean.
Data exclusions	No data was excluded.
Replication	Each experiment was repeated at least 3 times and with at least 3-5 animals in each group.
Randomization	Tumor-bearing mice were randomized following tumor implantation. For OC patients, subjects were randomized into 3 different treatment cohorts.
Blinding	Blinding in OC patient clinical trial was not possible due to need to give specific treatment in different treatment regimen. For mouse experiments, treatments were given in a blinded fashion by another investigator who was not involved in the treatment planning.

Reporting for specific materials, systems and methods

We require information from authors about some types of materials, experimental systems and methods used in many studies. Here, indicate whether each material, system or method listed is relevant to your study. If you are not sure if a list item applies to your research, read the appropriate section before selecting a response.

Materials & experimental systems

n/a	Involved in the study	
☐	☒	Antibodies
☐	☒	Eukaryotic cell lines
☒	☐	Palaeontology and archaeology
☐	☒	Animals and other organisms
☐	☒	Human research participants
☐	☒	Clinical data
☒	☐	Dual use research of concern

Methods

n/a	Involved in the study	
☒	☐	ChIP-seq
☐	☒	Flow cytometry
☒	☐	MRI-based neuroimaging

Antibodies

Antibodies used	Anti-VEGF antibody from Absolute Antibody, UK. For OC patients, bevacizumab was used.
Validation	Both antibodies have been used widely in animal studies or in clinical studies.

Eukaryotic cell lines

Policy information about cell lines

Cell line source(s)	ID8 tumor cell line from a collaborator.
Authentication	We tested the expression of reported tumor-associated antigens on this cell line and noting the in vitro culture doubling rated and morphology of the cell.
Mycoplasma contamination	No mycoplasma contamination detected. We conducted mycoplasma testing of this cell line regularly.
Commonly misidentified lines (See ICLAC register)	Not applicable.

Animals and other organisms

Policy information about studies involving animals; ARRIVE guidelines recommended for reporting animal research

Laboratory animals	Female, C57BL/6 mice
Wild animals	No wild animals used.
Field-collected samples	Not applicable
Ethics oversight	Animal protocols are approved by the Veterinary Service of Canton Vaud, Switzerland.

Note that full information on the approval of the study protocol must also be provided in the manuscript.

Human research participants

Policy information about studies involving human research participants

Population characteristics	Ovarian cancer patients with recurrent ovarian, fallopian tube, or primary peritoneal cancer were enrolled in a phase I, single-center study comprised of various sequential cohorts (NCT01132014). Patients' details are listed in Table 1.
Recruitment	Patients wwith recurrent ovarian, fallopian tube, or primary peritoneal cancer were enrolled in a phase I, single-center study comprised of various sequential cohorts (NCT01132014). Additional inclusion criteria required that patients were ≥18 years old, had sufficient tumor previously harvested at secondary cytoreductive surgery for lysate preparation, had completed physician's choice chemotherapy after secondary debulking and had a baseline Eastern Cooperative Oncology Group (ECOG) performance status 0-1.
Ethics oversight	The study was approved by the US FDA (BB-IND-14269) and by the University of Pennsylvania's Institutional Review Board. All patients gave written informed consent before initiation of any study procedures.

Note that full information on the approval of the study protocol must also be provided in the manuscript.

Clinical data

Policy information about clinical studies

All manuscripts should comply with the ICMJE guidelines for publication of clinical research and a completed CONSORT checklist must be included with all submissions.

Clinical trial registration	Clinical trial number NCT01132014 - www. clinicaltrials.gov
Study protocol	Full trial protocol is available from the authors upon request.
Data collection	Study Start Date : May 2010<br>Actual Primary Completion Date : May 30, 2018<br>Actual Study Completion Date : May 30, 2018
Outcomes	The primary objective of the trial was to establish the safety and biological activity of OCDC vaccine and the combinatorial regimens.

Flow Cytometry

Plots

Confirm that:

- [x] The axis labels state the marker and fluorochrome used (e.g. CD4-FITC).
- [x] The axis scales are clearly visible. Include numbers along axes only for bottom left plot of group (a 'group' is an analysis of identical markers).
- [ ] All plots are contour plots with outliers or pseudocolor plots.
- [x] A numerical value for number of cells or percentage (with statistics) is provided.

Methodology

Sample preparation	Methods are detailed in the supplementary methods and materials section.
Instrument	BD Canto flow cytometer (Becton Dickinson), and a 5-laser BD Fortessa instrument equipped with the FACS Diva software.
Software	Pro CellQuest software, FlowJo v10.5 (FLOWJO.LLC) and SPICE 5.1 software.
Cell population abundance	For human samples, we evaluated whole PBMCs, CD3+, CD8+ and CD4+ T cells from the PBMC populations. For animals, we evaluted purified CD8+ T cells.

Gating strategy	Gating strategies are detailed in supplementary materials and methods sections, as well as in supplementary Figures S1, S2 and S6.

☒ Tick this box to confirm that a figure exemplifying the gating strategy is provided in the Supplementary Information.
