# Supplementary material for: Personalized cancer vaccine strategy elicits polyfunctional T cells and demonstrates clinical benefits in ovarian cancer
Source: NPJ Vaccines. 2021 Mar 15;6:36. doi: 10.1038/s41541-021-00297-5 (PMC7960755; doi:10.1038/s41541-021-00297-5)
Supplement: Supplementary file 2 — Supplementary Information [file 41541_2021_297_MOESM2_ESM.pdf]

Supplementary figure and figure legends

Supplementary Figure 1.

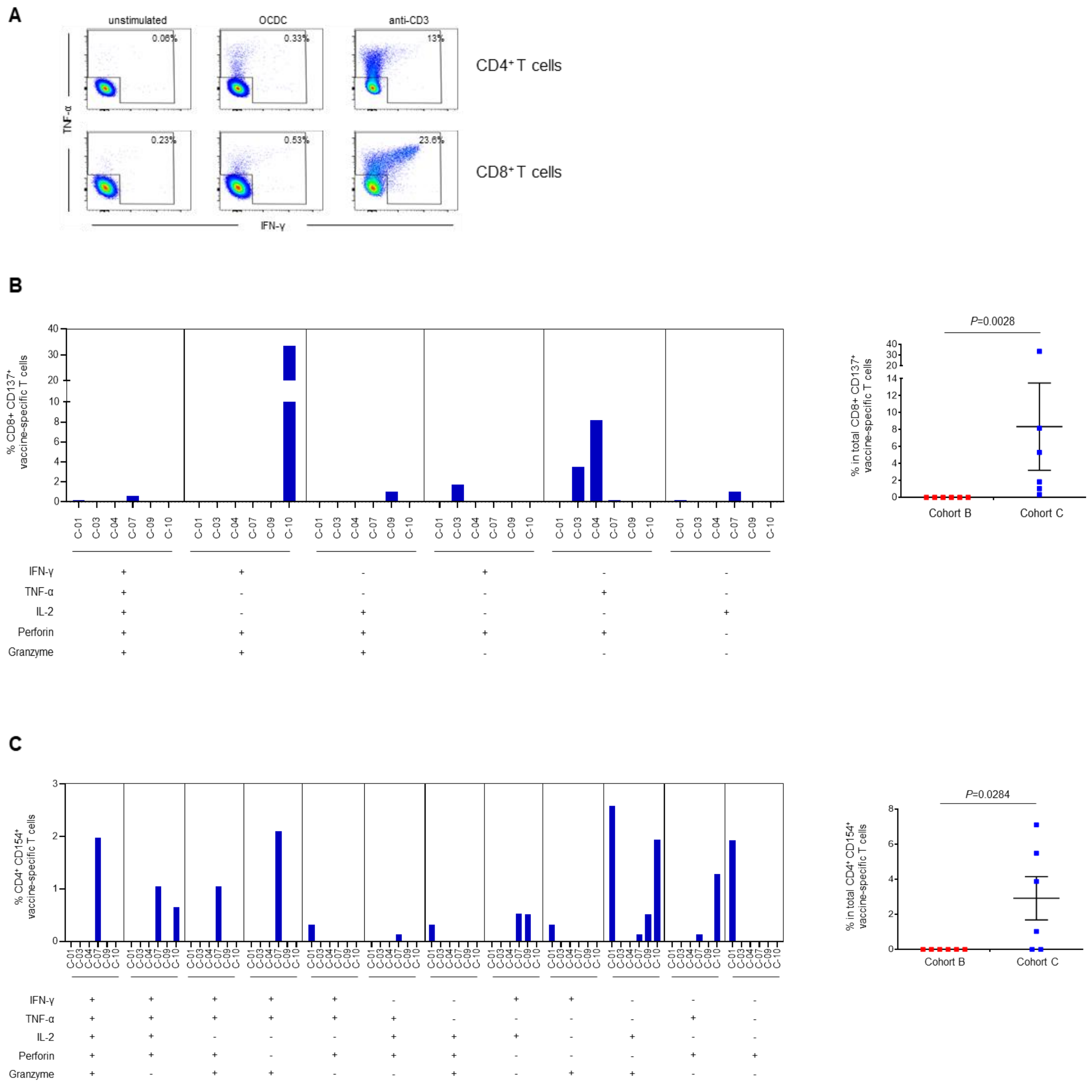

Supplementary Fig. 1 a Gating strategy demonstrated how OCDC vaccine-specific T cells were identified for downstream functional and phenotypic profiling showed in Fig. 2B. **b-c** Specific OCDC vaccine-specific $CD8^+$ (**b**) and $CD4^+$ (**c**) T cells that expressed IFN-γ, TNF-α, IL-2, perforin and/or granzyme B were detected in Cohort C and not in Cohort B patients (left panel). These $CD8^+$ and $CD4^+$ T cells were expressed as a percentage of the total elicited OCDC vaccine-specific $CD8^+CD137^+$ or $CD4^+CD154^+$ T cells in each Cohort C patient, and compared to Cohort B patients in Mann-Whitney test (right panel). $P<0.05$ was considered significant.

Supplementary Fig. 2

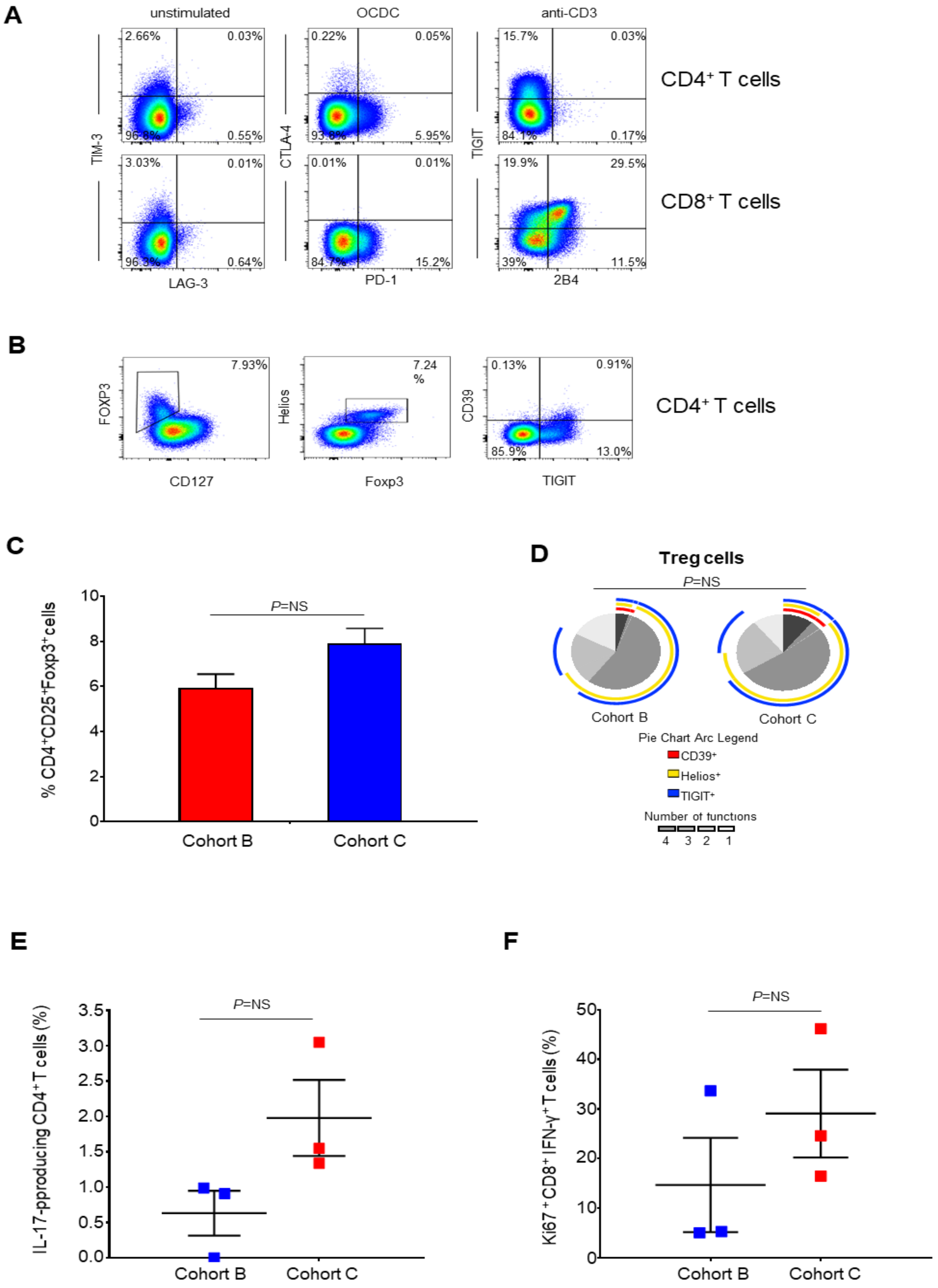

Supplementary Fig. 2 a Gating strategy demonstrating the different activation/exhaustion markers used to profile OCDC vaccine-specific $CD4^+$ and $CD8^+$ T cells showed in main **Fig. 2c**. **b** Gating strategy demonstrated how $CD4^+CD25^+Foxp3^+$ T reg cells were identified from $CD4^+CD127^-$ T cell population (left panel), and followed by further evaluation of Helio (middle panel), CD39 and TIGIT (right panel) expressions. **c** Percentage of $CD4^+CD25^+Foxp3^+$ cells in Cohort B and C patient at EOS. Results were reported as mean ± SEM. NS denoted that P>0.05 was not significant. **d** Cumulative functional profiling of Treg cells at EOS (co)expressing different functional markers as illustrated by the colored arcs. Pie charts were represented in shades of gray depicting the number of markers coexpressed in Cohort B and C patients. **e** Percentage of IL-17-producing OCDC vaccine-specific $CD4^+$ T cells in Cohorts B and C patients. **f** Percentage of $Ki67^+CD8^+$ T cells that were producing IFN-γ in responses to *ex vivo* OCDC stimulation in Cohort B and C patients. Vaccine-specific $CD4^+$ and $CD8^+$ T cells expressing the cytokines/marker above were identified as shown in main **Fig. 2a** in response to *ex vivo* OCDC stimulation. Results were reported as mean ± SEM. NS denoted that P>0.05 was not significant.

Supplementary Fig. 3

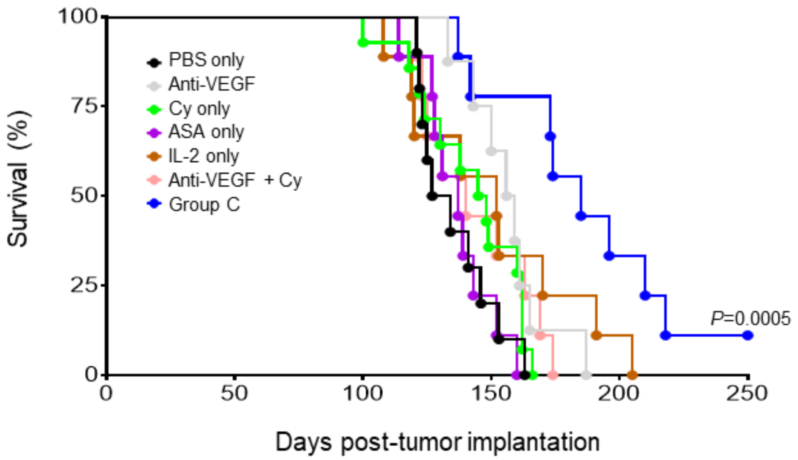

	P-value
Group C vs. PBS	0.0004
Group C vs. Anti-VEGF	0.022
Group C vs. Cy only	0.0006
Group C vs. ASA only	0.0006
Group C vs. IL-2 only	0.038
Group C vs. Anti-VEGF + Cy	0.0003
PBS vs. Anti-VEGF	0.023
PBS vs. Cy only	NS
PBS vs. ASA only	NS
PBS vs. IL-2 only	NS
PBS vs. Anti-VEGF + Cy	NS

Supplementary Fig. 3 Syngeneic 8-10 weeks old female C57BL/6 mice were implanted i.p. with ID8 tumor cells (5x10^6 cells/animal) and treated with monotherapy (i.e. ASA only, low-dose IL-2 only, low-dose Cy only), anti-VEGF+Cy or PBS following the Group C schedule as shown in main **Fig. 3a**. The survival curve of animals treated in Group C was compared to the survival curves of these treated animals (P=0.0005; Log-rank Mantel-Cox test). Group C showed the longest median OS. P<0.001 was considered highly significant, while P>0.05 was not significant. Total n=9 mice per group.

Supplementary Fig. 4

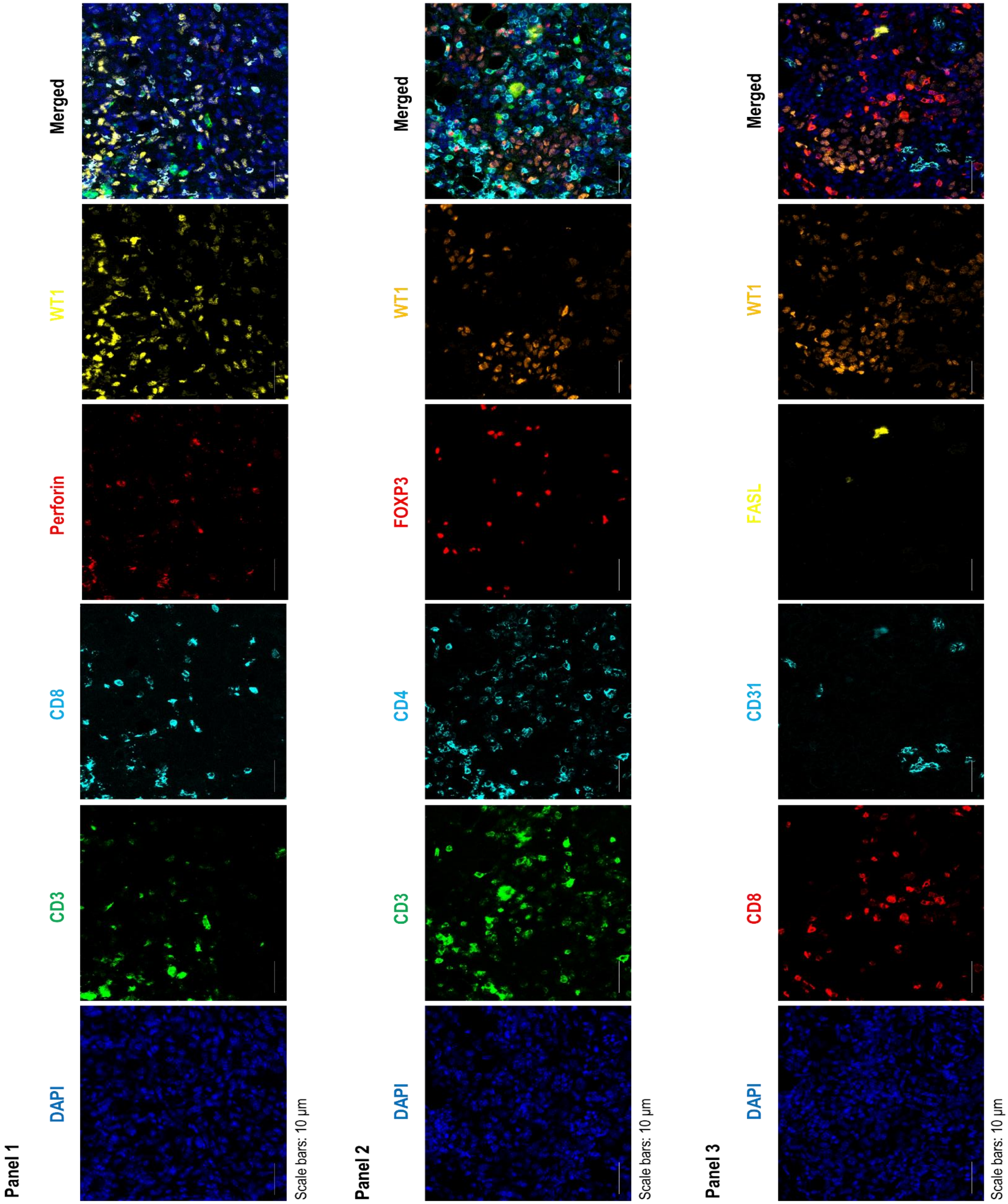

Supplementary Fig. 4 Immunohistochemistry (IHC) staining showing different immune and tumor markers in ID8 tumors (single color staining and merged). Panel 1 was used to determine TILs that expressed CD3, CD8 and perforin. Panel 2 was used to determine tumor-infiltrating Treg cells that expressed CD3, CD4 and Foxp3. Lastly, panel 3 was used to determine tumor endothelial cells expressing CD31 and FasL, as well as $CD8^+$ TILs. Staining with WT1 was performed to determine ID8 tumor cells expressing this tumor antigen, and DAPI was used to identify all nucleated cells in the tumor.

Supplementary Fig. 5

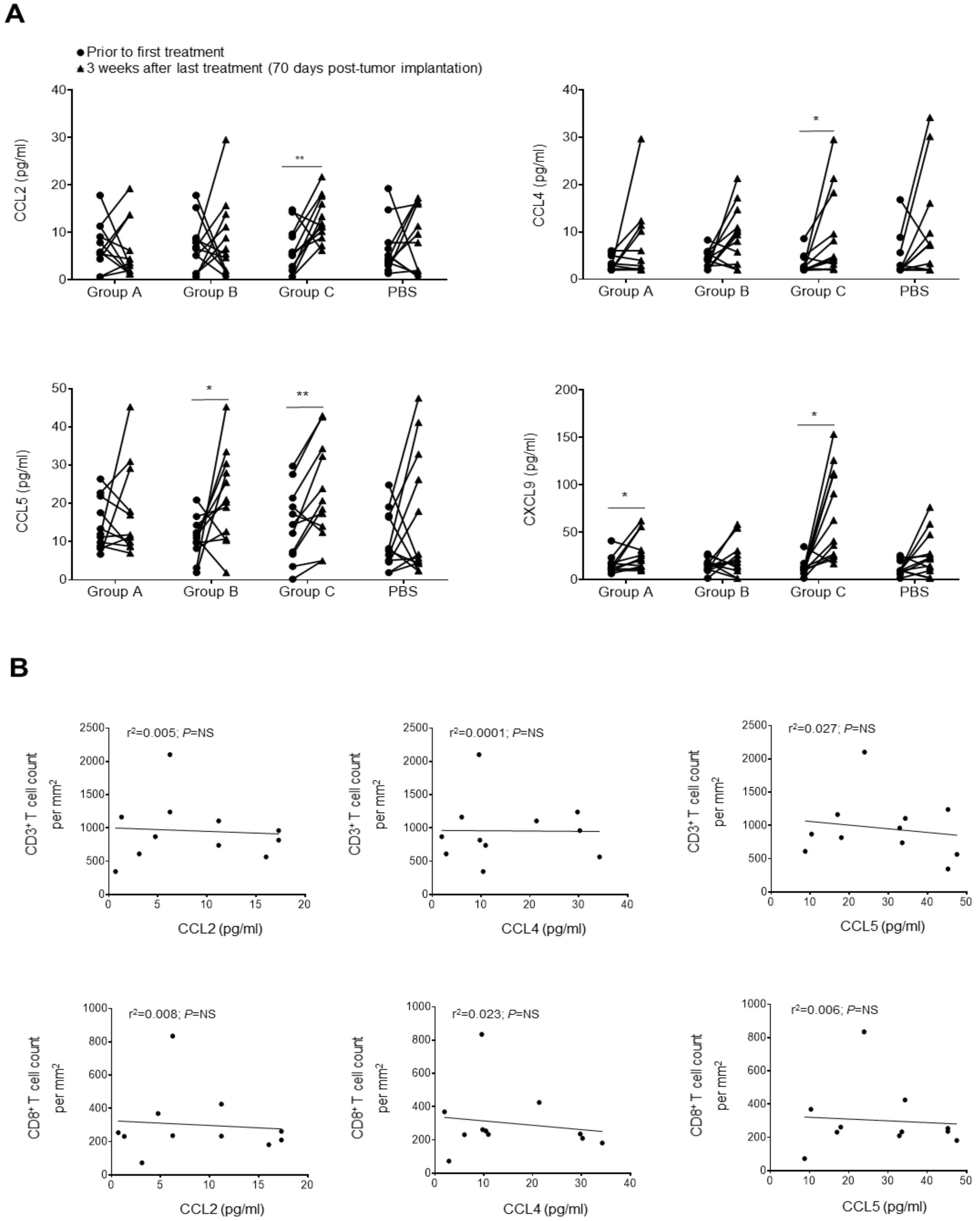

Supplementary Fig. 5 a Th1- and Th2-related plasma chemokine profiles in individual animals. Syngeneic 8-10 weeks old female C57BL/6 mice were implanted i.p. with ID8 tumor cells ($5x10^6$ cells/animal) and randomized into Group A, B or C. Blood was collected a day prior to the first treatment and 3 weeks after the last treatment, on day 19 and 70 post-tumor implantation, respectively. Plasma was evaluated for Th1- and Th2-related chemokines in a cytokine bead array assay. Each line corresponded to an animal and depicted a change in the plasma chemokine concentration prior to the first treatment and 3 weeks after the last treatment. Paired Student *t-test* was performed and the results reported as * or ** denoting significant *P*-values were <0.05 and <0.001, respectively. **b** Linear regression analysis to determine a positive correlation between CCL2, CCL4 and CCL5 with $CD3^+$ and $CD8^+$ TILs. P>0.05 was considered not significant. Total n=9 mice per group.

Supplementary Fig. 6

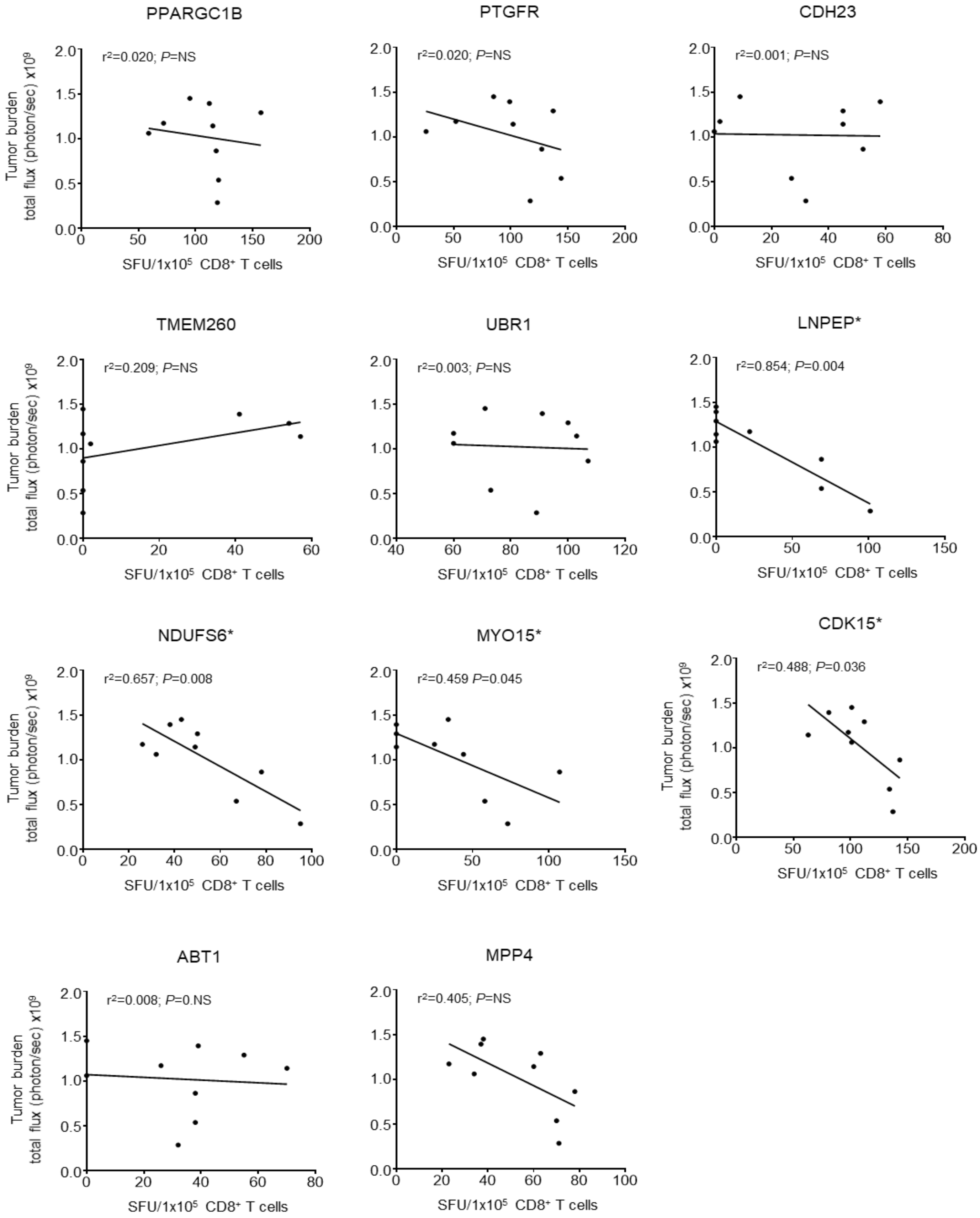

Supplementary Fig. 6 Linear regression analysis to determine a positive correlation between tumor burden at 3 weeks post-last treatment (bioluminescence total flux [photon per sec]x10^9) and the total number of IFN-γ spots elicited by individual neoantigen. *Astericks indicated that the neoantigen demonstrated a positive correlation result. $P<0.05$ was considered significant. Total n=9 mice per group.

Supplementary Fig. 7

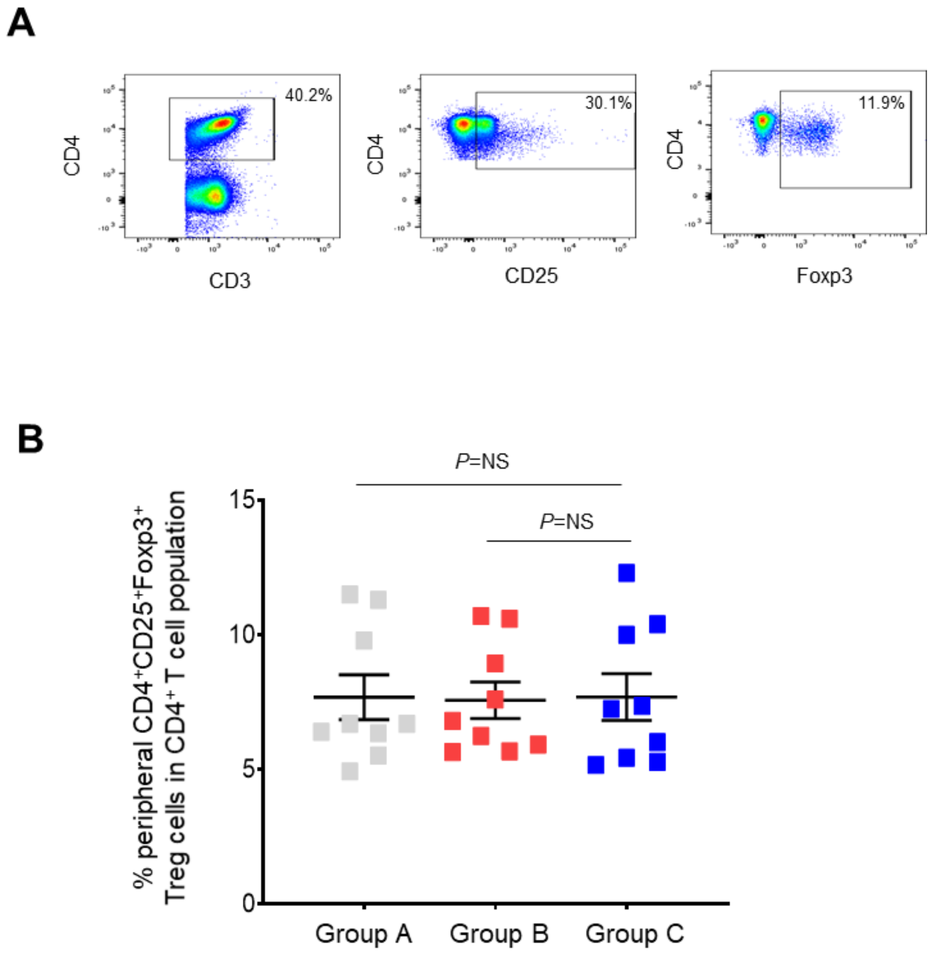

Supplementary Fig. 7 **a** Gating strategy demonstrated how mouse peripheral $CD4^+$ T cells were first identified from whole $CD3^+$ T cell population (left panel), and followed by CD25 expression (middle panel) and finally gated for Foxp3 expression (right panel). **b** Percentage of peripheral $CD4^+CD25^+Foxp3^+$ cells in Group A, B and C animals at 3 weeks post-treatment (70 days post-tumor implantation). Results were reported as mean ± SEM. NS denoted that P>0.05 was not significant.

Supplementary Table 1. List of neoantigens epitopes derived from patient B-08.

Supplementary Table S1. List of neoantigen epitopes derived from patient B-08.

	Gene	Entrez gene name	Mutation	Mutant Sequence	Number of amino acids (a.a)	Molecular weight (Da)
peptide pool	HIVEP3	Human Immunodeficiency Virus Type I enhancer binding protein 3	p.Pro2115Gln	QPAPLPHKL	9	1000.19
	PRRC2B	Proline rich coiled-coil 2B	p.Leu784Val	NESSFSASVGRAGGVS	16	1511.54
	ZW10	Zw10 kinetochore protein	p.Lys449Glu	VQEVSNTQYHEVMN	14	1677.78
	MYLPF	Myosin light chain, phosphorylatable, fast skeletal muscle	p.Val17Ile	GSSSIFSMF	9	962.07
	RBM22	RNA binding motif protein 22	p.Met387Ile	PPPGFGPHMFHPIGPP	16	1681.95
	GCFC2	GC-rich sequence DNA-binding factor 2	p.Gly250Arg	KIIEERDIDLSCGNR	15	1760.96
	TK1	Thymidine kinase 1	p.Arg41Ser	STELMRRVSRFQIAQ*	15	1822.09
	DISP2	Dispatched RND transporter family member 2	p.Ala867Val	DFPWVPQFF	9	1182.32
	PLXNA2	Plexin A2	p.Glu1330Asp	FPGIDDHPVL	10	1109.23
	HIVEP3	HIVEP zinc finger 3	p.Pro2115Gln	LDPRVLFQPAPLPH	14	1599.87
	UNC79	Unc-79 homolog, NALCN channel complex subunit	p.Pro164Thr	IFYTTTCLLTF	11	1322.56
	KCNJ12	Potassium inwardly rectifying channel subfamily J member 12	p.Asp402Glu	REGLSPQAR	9	1013.1
	CSMD2	CUB and Sushi multiple domains 2	p.Trp2169Leu	CQHGTNRNL	9	1042.13

* Indicated that the neoepitope was validated against its wild type sequence in Fig. 2D.

Supplementary Table 2. List of 17 immunogenic neoantigen epitopes derived from the murine ID8 ovarian tumor cell line.

Supplementary Table S2. List of 17 immunogenic neoantigen epitopes derived from the murine ID8 ovarian tumor cell line.

Protein	Entrez gene name	H-2 allele	Mutated sequence			Wild-type sequence	
			sequence	*In silico* affinity (nM)	*In vitro* affinity (O.D)	sequence	*In silico* affinity (nM)
PPARGC1B	Peroxisome proliferative activated receptor, gamma, coactivator 1 beta	$H\text{-}2\text{-}D^b$	RAMVQLI**H**YM	626.8	0.584	RAMVQLIRYM	1588.8
PTGFR	Prostaglandin F receptor	$H\text{-}2\text{-}D^b$	SMVFSGL**W**PL	1447.4	0.327	SMVFSGLCPL	2295.8
CDH23	Cadherin 23	$H\text{-}2\text{-}D^b$	FQPPS**L**FFAI	5241.6	0.523	FQPPSPFFAI	6935
TMEM260	Transmembrane protein 260	$H\text{-}2\text{-}K^b$	L**H**YLHYCEGL	105.2	1.172	LRYLHYCEGL	470.7
UBR1	Ubiquitin protein ligase E3 component n-recognin 1	$H\text{-}2\text{-}K^b$	V**S**AVLAFPSL	173.6	0.731	VGAVLAFPSL	281.5
LNPEP	Leucyl/cystinyl aminopeptidase	$H\text{-}2\text{-}K^b$	IT**D**ALFQTNL	174.8	0.732	ITEALFQTNL	182.9
NDUFS6	NADH:ubiquinone oxidoreductase core subunit S6	$H\text{-}2\text{-}K^b$	**A**LTFRRLLTL	241.3	0.370	VLTFRRLLTL	71.8
MYO15	Myosin XV	$H\text{-}2\text{-}K^b$	LGNYIVHQ**R**L	365.6	0.205	LGNYIVHQGL	465.3
CDK15	Cyclin-dependent kinase 15	$H\text{-}2\text{-}K^b$	AL**V**HDYFSVL	380.8	0.211	ALIHDYFSVL	357.2
ABT1	Activator of basal transcription 1	$H\text{-}2\text{-}K^b$	**C**LHRFTWSHL	736.5	0.674	YLHRFTWSHL	373.8
MPP4	Membrane protein, palmitoylated 4 (MAGUK p55 subfamily member 4)	$H\text{-}2\text{-}K^b$	IS**N**LTICAGL	739.9	0.320	ISDLTICAGL	825.6
ANKRD17	Ankyrin repeat domain 17	$H\text{-}2\text{-}D^b$	YACENG**Q**TDV	925.2	0.820	YACENGHTDV	560.7
SFI1	Sfi1 homolog, spindle assembly associated (yeast)	$H\text{-}2\text{-}D^b$	**R**AWSMWRECL	5262.4	0.284	WAWSMWRECL	5014.9
SFI1	Sfi1 homolog, spindle assembly associated (yeast)	$H\text{-}2\text{-}D^b$	SLPRDLDP**Q**L	6745.3	0.273	SLPRDLDPHL	6595.4
TLE1	Transducin-like enhancer of split 1	$H\text{-}2\text{-}D^b$	AGL**Y**SMSPQM	6063.9	0.299	AGLHSMSPQM	9040
ZFP169	Zinc finger protein 169	$H\text{-}2\text{-}D^b$	VAFSQ**T**EWEL	6986.4	0.650	VAFSQMEWEL	4787.8
FRY	FRY microtubule binding protein	$H\text{-}2\text{-}K^b$	R**G**FLFPQQSL	129.7	1.110	RRFLFPQQSL	1184.6
